# Chiral molecular imprinting-based SERS detection strategy for absolute enantiomeric discrimination

**DOI:** 10.1038/s41467-022-33448-w

**Published:** 2022-09-30

**Authors:** Maryam Arabi, Abbas Ostovan, Yunqing Wang, Rongchao Mei, Longwen Fu, Jinhua Li, Xiaoyan Wang, Lingxin Chen

**Affiliations:** 1 CAS Key Laboratory of Coastal Environmental Processes and Ecological Remediation, Yantai Institute of Coastal Zone Research, Chinese Academy of Sciences, 264003 Yantai, China; 2 Laboratory for Marine Biology and Biotechnology, Pilot National Laboratory for Marine Science and Technology, 266237 Qingdao, China; 3 School of Pharmacy, Binzhou Medical University, 264003 Yantai, China; 4 Center for Ocean Mega-Science, Chinese Academy of Sciences, 266071 Qingdao, China

**Keywords:** Sensors, Sensors and biosensors

## Abstract

Chiral discrimination is critical in environmental and life sciences. However, an ideal chiral discrimination strategy has not yet been developed because of the inevitable nonspecific binding entity of wrong enantiomers or insufficient intrinsic optical activities of chiral molecules. Here, we propose an “inspector” recognition mechanism (IRM), which is implemented on a chiral imprinted polydopamine (PDA) layer coated on surface-enhanced Raman scattering (SERS) tag layer. The IRM works based on the permeability change of the imprinted PDA after the chiral recognition and scrutiny of the permeability by an inspector molecule. Good enantiomer can specifically recognize and fully fill the chiral imprinted cavities, whereas the wrong cannot. Then a linear shape aminothiol molecule, as an inspector of the recognition status is introduced, which can only percolate through the vacant and nonspecifically occupied cavities, inducing the SERS signal to decrease. Accordingly, chirality information exclusively stems from good enantiomer specific binding, while nonspecific recognition of wrong enantiomer is curbed. The IRM benefits from sensitivity and versatility, enabling absolute discrimination of a wide variety of chiral molecules regardless of size, functional groups, polarities, optical activities, Raman scattering, and the number of chiral centers.

## Abstract (web summary)

Absolute chiral discrimination in chiral imprinted systems is complicated by the nonspecific binding of enantiomers. Here, the authors report a SERS “inspector” recognition mechanism to distinguish between specifically and nonspecifically bound enantiomers, even in seawater and urine.

## Introduction

Chirality is a ubiquitous phenomenon in nature. The enantiomers of a chiral molecule despite the similar physical and chemical properties, exhibit distinct pharmacological, biological, and toxicological activities. As an illustration, the toxic isomer of a racemic drug can induce severe side effects and genetic diseases that may cause death^1^. Chiral discrimination is an indispensable tool that has pivotal importance in accurately recognizing enantiomers^2–4^. An ideal chiral discrimination strategy must be realized by absolute enantiomeric discrimination in racemates, high recognition sensitivity, and be enforceable for wide varieties of chiral molecules regardless of molecular properties. So far, various techniques have been developed for enantiomeric discrimination that operated based on two main recognition mechanisms. First, methods based on intrinsic optical activities of chiral molecules in which a pair of enantiomers are distinguished through light–matter interactions^5–7^. Regrettably, these methods lack sufficient sensitivity and/or recognition performance because of the subwavelength molecular dimension, especially in systems with faint chiroptical signals, weak absorption bands, and trace chirality. For instance, the actual application of surface-enhanced Raman optical activity with high reproducibility and few spectral artifacts is still questioned because of high optical losses that can induce large photothermal heat. The photothermal heat aggravates the instability of the Raman optical activity signal, changes in dielectric properties of the nanoparticles, and even destroys the conformation of chiral molecules^8,9^. The other systems rely on chiral selectors, such as cyclodextrins^10^, metal surfaces with localized asymmetric sites^11^, covalent organic frameworks^12^, polymeric materials^13^, and chiral imprinted surfaces^14^. Host–guest interaction is the dominant mechanism of chiral discrimination in most such systems. By functionalizing the artificial interface with chiral hosts, chirality is initially introduced onto artificial interfaces. Then, a particular enantiomer of the chiral guests binds to the chiral interface via enantioselective intermolecular interactions, such as π–π interaction, hydrogen bonding, hydrophobic forces and translate the chirality information to the detectable signals. Although these approaches have achieved high sensitivity due to the participation of sensitive detection schemes such as fluorescence^15^, surface-enhanced Raman scattering (SERS)^16^, electrochemical^17^, and nuclear magnetic resonance^18^, they only work for a limited group of chiral compounds with specific physicochemical properties (e.g. Raman scattering^19^, charge^20^, viscosity^21^), thus detection versatility is an unmet need. More importantly, recognition resolution and molecular specificity information are absent due to the poor enantiospecificity of chiral selector units. The enantioselectivity value (*I*_good-enantiomer_/*I*_wrong-enantiomer_), a factor used to evaluate the discrimination performance, is only up to <3.0 for most reported works^22^, which is far from absolute chiral discrimination. All these defects suggest a dearth of an ideal chiral discrimination system is felt.

SERS is a highly powerful, functional, and versatile analytical detection technology that provides single-molecule-level sensitivity and high molecular fingerprint specificity^23,24^. However, discrimination of chiral compounds via traditional SERS substrates cannot be realized. Meanwhile, chiral imprinted systems (CISs) with the “lock-and-key” molecular recognition mechanism have been broadly developed, and an extensive variety of chiral binding sites have been imprinted into different materials^25–28^. To achieve both enantioselectivity and enantiosensitivity, the integration of CISs with SERS should emerge and possess immense potentials, by exploiting both SERS’ and CISs’ key advantages. However, research on developing CIS-SERS platforms for chiral discrimination has not been done yet due to the lack of a versatile and robust sensing mechanism. Irrespective of the types of target analytes (chemical or biological compounds), current sensing mechanisms for molecularly imprinted polymer-based SERS (MIP-SERS) platforms can be divided into two main categories^29^: (1) Selective capturing of analyte and direct sensing^30^. The potential application of this strategy for chiral discrimination is limited because most chiral compounds exhibit only weak Raman scattering resulting in a low sensitivity; enantiomers of a chiral molecule produce identical SERS signals and thereby cause data misinterpretation. Moreover, the direct chiral SERS sensing strategy is susceptible to matrix interferences, because strong SERS signals of the matrix compounds mask the analyte’s readout. (2) Labeling mode with the aid of SERS nanotags^31^. Although this strategy offers high sensitivity since it is independent of the Raman scattering of target species and is efficient for the detection of macromolecules with weak Raman scattering, it cannot be extended to chiral molecules. This methodology only works for macromolecules (e.g. proteins) with multiple binding sites, leastwise two sites, one site for interaction with the imprinted cavity of MIP, and the other site for the recognition unit of the SERS tag. As for small organic molecules such as almost all chiral compounds, their labeling with SERS tags is impossible. Even if by modification of this strategy labeling of captured enantiomer molecules in the MIP can be realized, creating asymmetric adsorption sites on the SERS tags surface to provide chiral environments for enantiomers to adsorb differently will be essential, but this will make the method sophisticated. Additionally, high susceptibility to matrix interferences from other species that can induce similar SERS tags signal change is the other crucial drawback of this approach. Hence, for analyzing complex real samples, implementation of sample preparation (which commonly involves centrifugation, pH adjustment, etc.) is required.

Therefore, a robust sensing strategy, which can be implemented on CIS-SERS platforms, is demanded to realize absolute chiral discrimination of wide varieties of chiral compounds. To realize this aim, the occurrence of nonspecific recognition in the CISs is the main barrier. In principle, the size, shape, and functionality of the target molecule contribute to the molecular complementary recognition mechanism^14,32,33^. Enantiomers of a chiral molecule have identical size and functionality, differing only in the spatial arrangement of one or more chiral center(s). In practice, the enantioselectivity of generated chiral imprinted cavities merely derives from the stereostructure discrepancy. This phenomenon leads to the acute nonspecific binding entity in all CISs and a lack of achieving absolute enantiospecific discrimination^34–36^. Hence, chirality information obtained from CISs is not absolute enantiospecific due to the participation of nonspecific recognition of wrong enantiomers in chiral discrimination^37–39^. In other words, the wrong enantiomer unavoidably generates a signal that leads to inaccuracy of the results. Two rational strategies that improved the stereoselectivity of CISs include the following: (1) developing advanced chiral imprinting approaches, including imprinting in a mesoporous metal matrix^14^ and chiral imprinting of a helical micelle^40^; (2) controlling the interactions in chiral recognition^20^. Unfortunately, neither of these strategies can render absolute enantiospecific discrimination in practice, and research on developing new strategies that are able to distinguish specific recognition from nonspecific recognition has remained in infancy. The main reason for this research gap is that the scrutiny of the spatial position of bonded chiral molecules and imprinted cavities of CISs on the molecular scale is very challenging to ascertain the nature of recognition.

Here, we move forward in five important aspects. First, the origin of nonspecific recognition of the wrong enantiomer in CISs that prevents absolute chiral discrimination is systematically identified. Second, we propose a SERS-based inspector recognition mechanism (IRM) in which after chiral recognition, the small and linear shape inspector molecule scrutinizes the status of chiral imprinted cavities and precisely discerns the specific binding of good enantiomers; thus, absolute enantiospecific discrimination is realized in the CISs. Third, we show that the IRM is versatile as exemplified by the discrimination of six chiral amino acids and three monosaccharides with different sizes, functional groups, polarities, and the number of chiral centers. Fourth, the developed IRM is highly sensitive. The LODs reach down to ng L^−1^, which are lower by at least four orders of magnitude than the reported values for chiral amino acids. Also, enantiomeric excess (ee) can be measured at the ultratrace level. Finally, accurate enantiorecognition of chiral compounds in complex environmental and biological samples is realized.

## Results and discussion

### Principle of the IRM

**Fig. 1:**
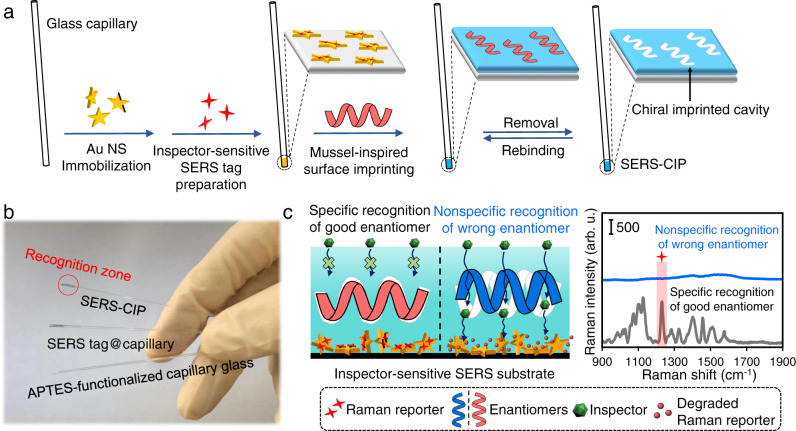
The SERS-CIP construction and principle of IRM. **a** Schematic illustration of the SERS-CIP construction. **b** Photo images of APTES-functionalized capillary glass, SERS tag@capillary, and SERS-CIP. The recognition zone is illustrated by a red circle. **c** Principle of IRM implemented on SERS-CIP.

The IRM is executed on a SERS chiral imprinted platform (SERS-CIP) that consists of two distinct layers immobilized on the outer surface of the glass capillary (Fig. 1a, b): outer chiral imprinted polydopamine (PDA) rich in chiral cavities, which is responsible for chiral recognition. And an inner inspector-sensitive SERS tag^41^, whose signal change is used to ascertain the imprinted cavities’ filling status. The IRM consists of two steps. First, chiral molecules are recognized by imprinted cavities of the PDA layer. Once recognition occurs (both specific and nonspecific), imprinted cavities of PDA are only fully occupied by the good enantiomer (perfect fit, Fig. 1c). Second, the spatial status of chiral imprinted cavities and bonded enantiomers is scrutinized by an inspector based on a blocking mechanism. In the IRM, the PDA network is impermeable to the inspector flow, and chiral imprinted cavities within PDA are the exclusive inspector pathway to inspector-sensitive SERS substrates. Therefore, the access of the inspector to a portion of the inspector-sensitive SERS substrate is blocked under the specifically filled cavities. In the nonspecific recognition event, the inspector freely attaches to inspector-sensitive SERS substrate through vacant or nonspecifically occupied cavities and induces SERS signal suppression by degradation of Raman reporter molecules.

Three key points resulting in the successful implementation of the IRM are as follows: (1) Imprinted PDA is used as a universal chiral recognition unit. PDA has many noncovalent functionalities such as hydroxy-containing and amino-containing groups as well as π–π bonds^42,43^ that exactly match the characteristics of imprinted materials for efficient recognition of a wide variety of chiral molecules. Additionally, amine and phenolic hydroxyl groups make PDA potentially ampholytic or zwitterionic. Hence, PDA exhibits fully reversible, pH-switchable permselectivity for both cationic and anionic molecules^44^. Dopamine can be readily oxidized and polymerized in both aerobic alkaline and acidic media at room temperature. Therefore, imprinting of thermo and pH-sensitive species can be easily implemented. Most importantly, PDA density/thickness, which determines the permeability to the inspector, can be precisely adjusted by changing polymerization variables. (2) Homogenous inspector-sensitive SERS active substrates composed of gold nanostars (Au NSs) and 3.3’-diethylthiatricarbocyanine iodide (DTTC) Raman reporters. This couple achieves sensitive enhancement under 780 nm laser irradiation and assures the sensitivity of the IRM. (3) Linear shape inspector molecule. The scrutiny of chiral imprinted cavities by an inspector molecule, resulting in curbing nonspecific recognition of wrong enantiomers. The inspector molecule can only percolate through unoccupied imprinted cavities left by wrong enantiomer recognition, and quench the signal of a portion of the SERS substrate located under these cavities by chemical degradation of DTTC. Meanwhile, correctly occupied imprinted cavities stem from specific recognition of good enantiomer are impermeable to the inspector and SERS signal of DTTC remains unchanged. Accordingly, chiral discrimination information is translated.

### Study of nonspecific recognition by CISs

**Fig. 2:**
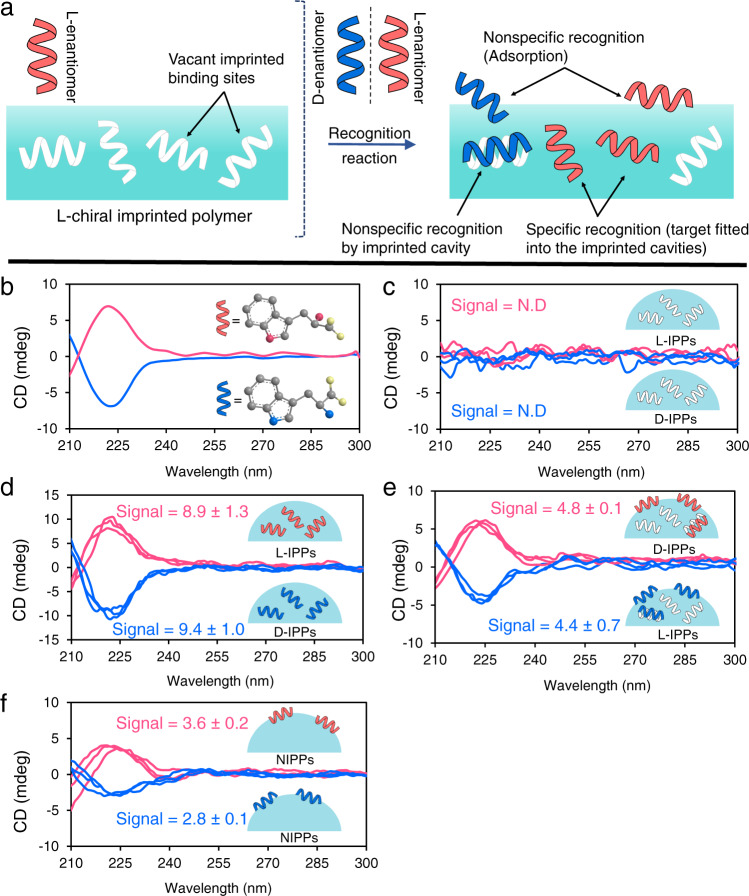
Study of non-specific recognition on the CIS. **a** Status of chiral molecules and imprinted cavities of CISs after a recognition event. CD spectra of **b** standard solution of l-tryptophan and d-tryptophan, **c** l-IPPs and d-IPPs, **d** l-IPPs and d-IPPs after recognition of l-tryptophan and d-tryptophan, respectively, **e** l-IPPs and d-IPPs after recognition of d-tryptophan and l-tryptophan, respectively, **f** NIPPs after recognition of l-tryptophan and d-tryptophan. Pink and blue lines are related to l-tryptophan and d-tryptophan, respectively. The inset in “**b**” depicts the molecular structure of tryptophan enantiomers. The insets in “**c**–**f**” are bonded enantiomers’ statuses on the receptors. Standard deviations were obtained from three measurements. ND not detected. Source data are provided as a Source Data file.

Nonspecific recognition (nonfitting geometry) unavoidably participates in chiral recognition and stems from the interactions among functional groups of polymer frameworks and chiral molecules^45^, leading to unwanted binding of wrong enantiomers in imprinted cavities and surface adsorption of chiral molecules (Fig. 2a). To experimentally verify this effect, chiral imprinted polydopamine particles (CIPPs) and nonimprinted polydopamine particles (NIPPs), which are more readily characterized by circular dichroism (CD) spectroscopy, Fourier transform infrared (FT-IR) analysis, and isothermal titration calorimetry than SERS-CIP, were synthesized. The interactions with the chiral guest model (tryptophan) were explored by the three techniques. l-tryptophan and d-tryptophan possess positive and negative CD signals at ~220 nm, respectively (Fig. 2b). CIPPs before chiral recognition lack CD opposite signals (Fig. 2c). After tryptophan enantiomer recognition by CIPPs, intense positive and negative CD signals corresponding to l-tryptophan and d-tryptophan, respectively, were observed (Fig. 2d). From Fig. 2e, wrong enantiomers can be captured by the l-IPPs and d-IPPs demonstrating nonspecific binding in chiral recognition. Although there was no chiral imprinted cavity into the NIPPs, the emergence of weak opposite CD signals in Fig. 2f revealed that both enantiomers of tryptophan were nonspecifically recognized by NIPPs through surface adsorption. Compared with that in the CD spectra of NIPPs, the signal intensity of l-IPPs and d-IPPs in their wrong enantiomers after the recognition reaction is higher, suggesting that nonspecific binding could occur in the chiral imprinted cavities in addition to surface adsorption. Moreover, from the results of intermolecular interaction (binding constant values of 5.4 × 10^5^, 1.2 × 10^6^, 1.7 × 10^5^, 1.5 × 10^4^, 3.6 × 10^5^, and 1.8 × 10^4^, for d-IPPs with d-tryptophan, l-IPPs with l-tryptophan, d-IPPs with l-tryptophan, l-IPPs with d-tryptophan, NIPPs with d-tryptophan, and NIPPs with l-tryptophan, respectively), CIPPs and NIPPs have a high binding affinity for wrong enantiomers, indicating that nonspecific binding has a great contribution to chiral recognition (see the Supporting Information for further binding data regarding tryptophan enantiomer guests with CIPPs and NIPPs). A study of the intrinsic nature of chiral recognition by FT-IR spectroscopy (by monitoring the intensity of the distinct peak of tryptophan at 744 cm^–1^), which is in accordance with CD results is presented in the Supporting Information.

### Working mechanism of inspector molecule

**Fig. 3:**
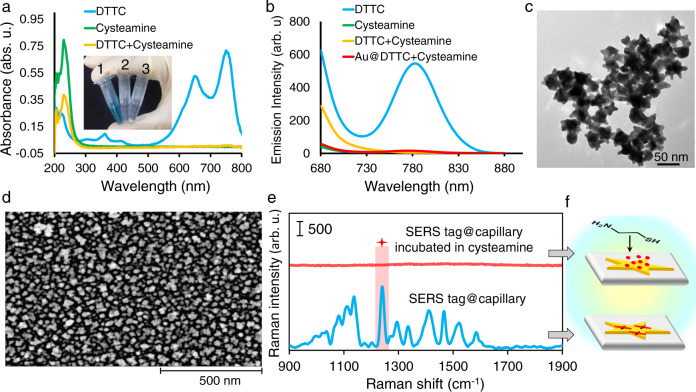
Chemical degradation of DTTC by an inspector molecule. **a** UV–Vis and **b** fluorescence spectra of the reactants and degradation products. **c** TEM image of Au NSs. **d** SEM images of Au NSs assembled on capillary glass. **e** Raman spectra of DTTC on different platforms and **f** the corresponding platform statuses. The inset in “**a**” is a photo image of (1) DTTC, (2) cysteamine, and (3) their mixture. Source data are provided as a Source Data file.

By considering the functional group and molecular size, cysteamine (a linear shape and small aminothiol molecule) was employed as an inspector to track the filling status of the chiral cavities. Cysteamine can break the π-conjugate connection between donor and acceptor electron units of DTTC, chemically degrade DTCC quickly, and suppress the SERS signal. The expected degradation reaction is shown in Supplementary Fig. 1, and the possible mechanisms are presented in Supplementary Fig. 2. Ultraviolet–visible (UV–Vis) and fluorescence spectra of DTTC, cysteamine, and their mixture revealed degradation products lacking UV–Vis absorbance and fluorescence emission (Fig. 3a, b). These observations confirmed that the degradation reaction is fast and irreversible. The prepared inspector-sensitive SERS tag layer comprising Au NSs and DTTC (Fig. 3c, d) provides an intense SERS signal (Fig. 3e). To verify the feasibility of the DTTC degradation reaction on the surface of Au NSs, the SERS signal of SERS tag@capillary incubated in cysteamine was detected. Under this condition, the signal completely vanished, demonstrating complete degradation of all DTTC molecules. The status of the platforms is demonstrated in Fig. 3f. It should be noted that cysteamine has no effect on the stability of the PDA layer or the interactions among bonded chiral molecules and PDA.

### Density and thickness of the chiral imprinted layer

In the IRM, the thickness and density of the PDA layer are important in two aspects. First, a highly cross-linked polymer layer should be established to conserve the chiral imprinted cavities after template removal. Second, to execute the IRM flawlessly, the exclusive pathway of the inspector to the SERS tag must be chiral imprinted cavities (the molecular size of cysteamine is lower than the size of imprinted cavities; hence, it can pass through the unoccupied or nonspecifically filled imprinted cavities). This process requires an impermeable polymer network for the inspector flow. The thickness of the PDA layer can be easily controlled by adjusting the imprinting time^46,47^. To find a sufficient density of the polymer layer, the permeability of PDA to the inspector during the imprinting process was studied. The optimal imprinting time was investigated by tracking SEM images and Raman spectra of the SERS-CIP and SERS nonimprinted platform (SERS-NIP), results demonstrated imprinting time of 3 h provided desirable imprinted layer thickness (see the Supporting Information). In the early hours of polymerization, the PDA layer is less cross-linked and there are vacant pores within the polymers that are large enough for cysteamine penetration. Additionally, part of the SERS tag surface is still bare and lacks PDA growth and coverage. With increasing polymerization time, the PDA layer becomes gradually denser and thicker and therefore impenetrable to cysteamine flux. After imprinting, due to template removal, different regions of the chiral imprinted PDA layer possess heterogeneous densities. Low-density zones are permeable to the inspector, which derive from the depletion of chiral template loci (imprinted cavities); high-density zones form portions of the 3D PDA network in which no imprinting has taken place. If the thickness and/or density is more than the optimal amount, cysteamine cannot access all imprinted cavities and inspect them, because they are buried in a highly dense polymer network (Supplementary Fig. 3).

### The status of platforms

**Fig. 4:**
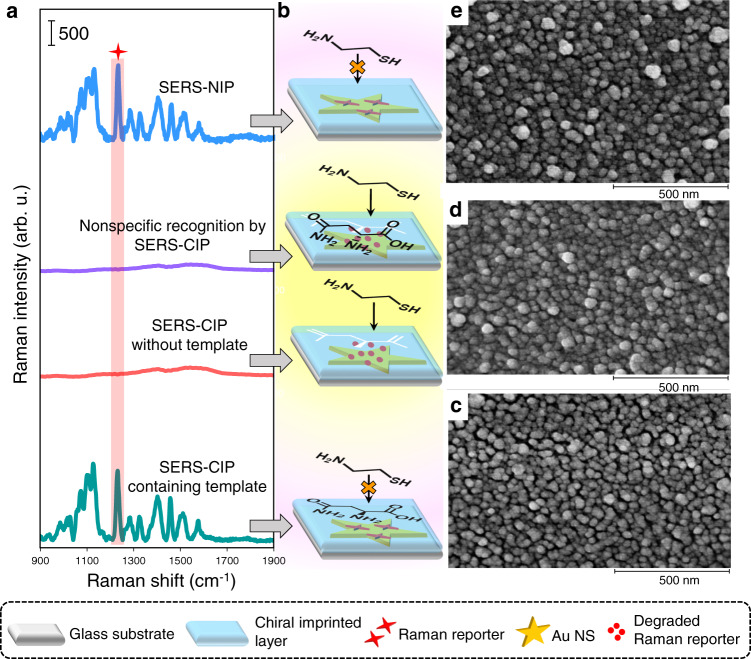
Implementation of the IRM on SERS-CIP. **a** Raman spectra of DTTC on different platforms, and **b** corresponding platform statuses. SEM images of **c** SERS-CIP containing a d-asparagine template, **d** SERS-CIP without a d-asparagine template, and **e** SERS-NIP (scale bar: 500 nm). The polymerization time was 3 h for the construction of the platforms. Source data are provided as a Source Data file.

The status of platforms in view of the IRM was surveyed. Figure 4 shows the Raman spectra (a), the corresponding platform status (b), and SEM images (c–e) of the optimal SERS-CIP and SERS-NIP. After coating the PDA layer containing the template (asparagine) on the SERS tag surface, the access of cysteamine to the SERS tag is interrupted, and the SERS intensity of DTTC remains unchanged. Removing template molecules from the PDA 3D network means initiating specific pathways (imprinted cavities) for cysteamine flux to the SERS tag destination. Accordingly, DTTC molecules degrade by reaction with cysteamine arriving from vacant imprinted cavities, thereby suppressing the SERS signal. After the recognition of the wrong enantiomer, imprinted cavities are not fully occupied, and cysteamine passes through them; therefore, the signal vanishes. There was no imprinted cavity in the SERS-NIP, and its SERS intensity remained unchanged after inspection by cysteamine. These observations corroborated that chiral imprinted cavities represent an exclusive pathway of cysteamine to access the SERS tag. Similar results were obtained for all SERS-CIPs constructed by using arginine, histidine, tryptophan, aspartic acid, alanyl-alanine, ribose, glucose, and galactose as the chiral models after relevant optimization. The diffusion rate of cysteamine within imprinted and nonimprinted PDA was investigated by collecting inspector incubation time-dependent SERS spectra (see the Supporting Information for details).

### The inspector molecular characteristics meet versatility

In the IRM an inspector molecule is responsible for scrutinizing the status of imprinted cavities after chiral recognition and reporting the binding information of imprinted cavities to an inspector-sensitive SERS tag layer. Both molecular size and functional groups of the inspector have pivotal roles in the successful implementation of the IRM. Hence, the selection of a suitable inspector molecule takes place based on three criteria as follows: (i) Its molecular size must be smaller than the size of chiral imprinted cavities to percolate through vacant or nonspecifically occupied cavities without spatial hindrance. (ii) It must not influence the interactions between bonded enantiomers and chiral imprinted cavities. Moreover, the chiral imprinted polymer should be chemically/physically stable during incubation in the inspector solution. (iii) It must degrade Raman reporter fast and irreversible. Linear shape aminothiol molecules can meet the requirements of (ii) and (iii). The molecular size of the inspector is tunable, meets the requirement of (i) criteria, and brings versatility to the mechanism. Almost all kinds of chiral molecules can be imprinted in the polymer network^48^. To implement IRM for different chiral targets, an inspector should be selected based on chiral molecular size and structure. Linear shape aminothiol molecules with different molecular sizes containing two to several methylene groups are commercially available.

**Fig. 5:**
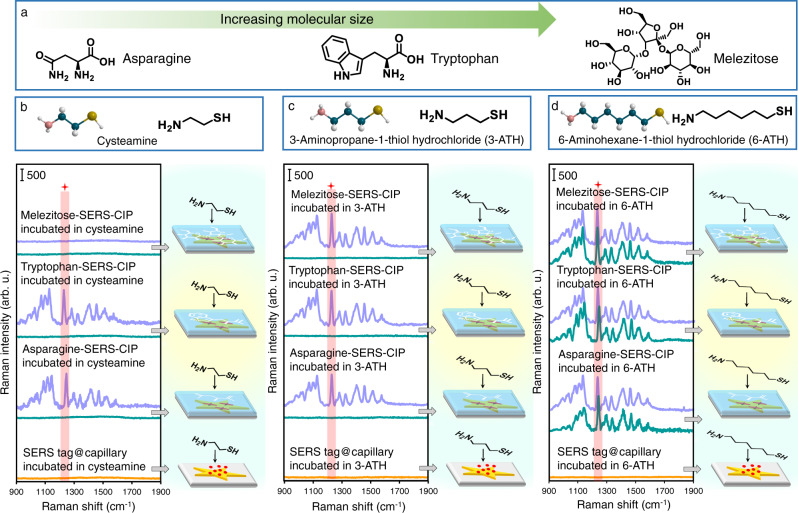
Versatility of the IRM. **a** Chemical structure of asparagine, tryptophan, and melezitose. Raman spectra of DTTC on different platforms, including SERS tag@capillary, asparagine-SERS-CIP, tryptophan-SERS-CIP, and melezitose-SERS-CIP, inspected by **b** cysteamine, **c** 3-ATH, and **d** 6-ATH. Green and purple colors demonstrate SERS-CIP incubated in blank solution and corresponding enantiomer solution with the concentration of 1000 µg L^−1^, respectively. Platform status of SERS-CIP incubated in blank solution before inspection is schematically illustrated in (**b**–**d**). Source data are provided as a Source Data file.

To investigate the influence of inspector size, three model SERS-CIP for tryptophan, asparagine, and melezitose target chiral compounds (Fig. 5a) were constructed and the IRM was implemented by using three inspector molecules with different sizes, including cysteamine, 3-aminopropane-1-thiol hydrochloride (3-ATH), and 6-aminohexane-1-thiol hydrochloride (6-ATH). 3-ATH and 6-ATH have linear shapes and functional groups (thiol and amine) similar to those of cysteamine but contain three and six methylene groups, respectively (Fig. 5b–d). Moreover, like cysteamine, 3-ATH and 6-ATH can degrade DTTC quickly. The results of cysteamine-based IRM revealed that the SERS signals of blank asparagine-SERS-CIP, tryptophan-SERS-CIP, and melezitose-SERS-CIP were completely quenched after inspection with cysteamine, demonstrating free percolation of cysteamine through vacant chiral imprinted cavities (Fig. 5b). After the recognition of target enantiomers on the corresponding SERS-CIP, the SERS signals the intensity of asparagine-SERS-CIP and tryptophan-SERS-CIP remained unchanged, whereas melezitose-SERS-CIP lacked signal. Melezitose has a larger molecular size than asparagine and tryptophan. The SERS analysis results clearly reveal that cysteamine can percolate through even specifically filled imprinted cavities of melezitose-SERS-CIP due to its too small size. Accordingly, a larger inspector molecule is required for the enantiorecognition and chiral discrimination of large-size chiral molecules by the IRM. At the next stage, the IRM was implemented on the SERS-CIP by using 3-ATH as an inspector (Fig. 5c). Similar to cysteamine-based IRM, none of the blank SERS-CIP exhibited SERS signal owing to the free penetration of 3-ATH through vacant imprinted cavities and complete chemical degradation of DTTC. Interestingly, after chiral recognition and inspection, the SERS signals of all SERS-CIP remained unchanged, suggesting that specifically occupied asparagine, tryptophan, and melezitose imprinted PDA layers are impermeable to the 3-ATH flux. Thus, 3-ATH is a suitable inspector for the inspection of large-size imprinted cavities (e.g., specific binding cavities complementary to melezitose). As expected, while 6-ATH was used as an inspector, the large size inspector could not penetrate through even vacant imprinted cavities and the intensity of the SERS signals of neither of the SERS-CIP was changed. This phenomenon occurs because the accessibility of 6-ATH to the SERS tag of all platforms is restricted due to the small size of imprinted cavities and spatial hindrance (Fig. 5d). These results illustrate the influence of an inspector’s molecular properties (size and functional groups), criteria for selecting a suitable inspector, and verifying the versatility of IRM.

### Chiral recognition and absolute enantiomeric discrimination

**Fig. 6:**
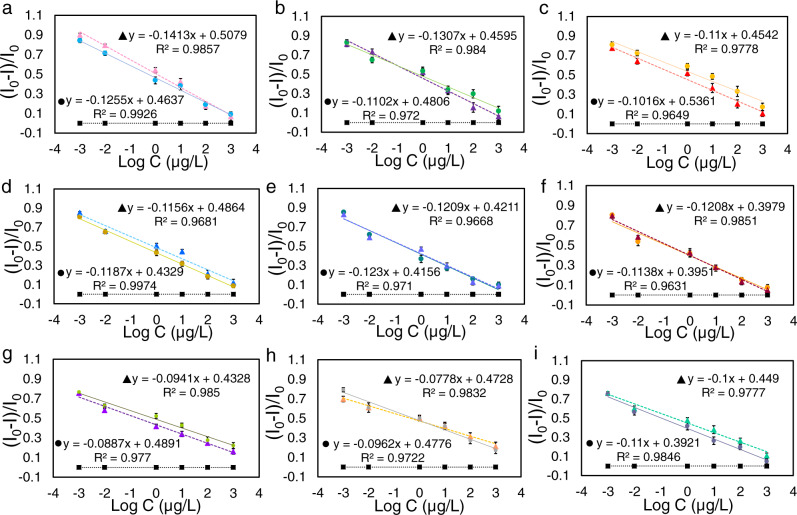
Response range of SERS-CIPs. Linear relationship between the relative SERS intensity ((*I*_0_−*I*)/*I*_0_) of SERS-CIP and the logarithm of enantiomers concentration. *I*_0_ and *I* are the SERS intensities of DTTC before and after IRM, respectively. **a** Arginine, **b** Asparagine, **c** Histidine, **d** Tryptophan, **e** Glucose, **f** Galactose, **g** Aspartic acid, **h** Alanyl-alanine, and **i** Ribose. ▲ and ● are related to d-enantiomers and l-enantiomers, respectively. ■ black lines are related to SERS-NIP. Raman signals of five different SERS-CIP, which were constructed in different batches, were analyzed for each data point. Error bars represent standard deviations. Source data are provided as a Source Data file.

For a proof of concept, enantiomers of five types of chiral amino acids, namely amino acids with positively charged side chains, arginine, and histidine; amino acids with negatively charged side chains, aspartic acid; amino acid with polar uncharged side chain, asparagine; aromatic amino acid with hydrophobic side chains, tryptophan; an amino acid with two chiral centers, alanyl-alanine; and three monosaccharides with four chiral centers, ribose, glucose, and galactose; as molecular units of numerous biological substances were recognized by the IRM at the ultratrace level. The corresponding SERS-CIP for each enantiomer was constructed, and the relevance between the number of enantiomers and the SERS intensity of DTTC after the IRM was examined. As expected, for the lowest amounts of enantiomers, the minimum SERS intensity was observed, and by the augmenting enantiomer quantity, the signals were evidently increased (Fig. 6a–i). Along with specific recognition, imprinted cavities of PDA were filled gradually and the cysteamine flow path was partially blocked by specifically bonded enantiomers; this process continued until complete blockage of all cysteamine flow. The change in SERS intensity was linearly dependent on the concentrations of enantiomers ranging from 0.001–1000 µg L^−1^ (corresponding Raman spectra are presented in the Supporting Information, Supplementary Fig. 4). The correlation coefficients and the linear regression equations are provided in Fig. 6a–i. The calculated detection limits were 2 × 10^−4^, 5.4 × 10^−4^, 6.3 × 10^−5^, 1.6 × 10^−4^, 1.1 × 10^−4^, 2.7 × 10^−5^, 5 × 10^−5^, 1.1 × 10^−4^, 5.1 × 10^−5^, 5.2 × 10^−5^, 1.4 × 10^−5^, 2.9 × 10^−5^, 1.9 × 10^−4^, 2.4 × 10^−4^, 3.5 × 10^−4^, 4.4 × 10^−4^, 5.6 × 10^−4^, and 2.3 × 10^−4^ µg L^−1^ for l-arginine, d-arginine, l-asparagine, d-asparagine, l-histidine, d-histidine, l-tryptophan, d-tryptophan, l-glucose, d-glucose, l-galactose, d-galactose, d-aspartic acid, l-aspartic acid, d-alanyl-d-alanine, l-alanyl-l-alanine, d-ribose, and l-ribose, respectively, which are the lowest values compared with those obtained by CD-, fluorescence-, and SERS-based detection (Supplementary Table 1). SERS-NIPs show almost identical signals for all tested enantiomers, which further corroborates that cysteamine is an exclusive inspector for these imprinted cavities.

**Fig. 7:**
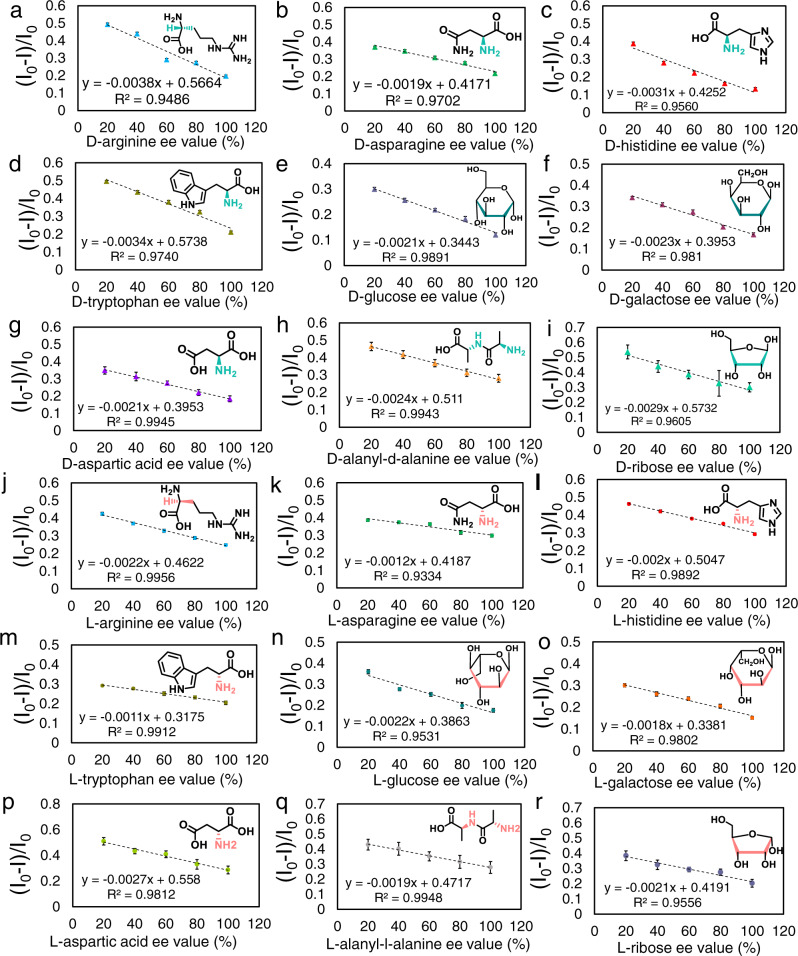
Chiral discrimination by the SERS-CIPs based on IRM. **a**–**h**, and **i** Linear relationship between the relative SERS intensity ((*I*_0_−*I*)/*I*_0_) and d-enantiomers in racemic mixtures with different ee % values after recognition with SERS-d-IP. **j**–**q**, and **r** Linear relationship between the relative SERS intensity ((*I*_0_−*I*)/*I*_0_) and l-enantiomers in the racemic mixture after recognition with SERS-l-IP. *I*_0_ and *I* are the SERS intensities of DTTC before and after IRM, respectively. Raman signals of five different SERS-CIP, which were constructed in different batches, were analyzed for each data point. Error bars represent standard deviations. Source data are provided as a Source Data file.

In the next stage, the enantiospecificity of the IRM was assessed in racemic mixtures with various ee. SERS-l-IPs and SERS-d-IPs were constructed for all enantiomers and used for the recognition of enantiomers in their corresponding racemic mixtures with a constant total concentration of 100 µg L^−1^ (different ee %). Figure 7a–r shows the responses of the SERS-d-IPs and SERS-l-IPs to the relative content of enantiomers, respectively. From Fig. 7a–i, by increasing the amount of d-enantiomers in the racemic mixtures, only specifically bonded d-enantiomers were recognized, and the responses of SERS-d-IPs decreased gradually, notwithstanding the constant total concentration of the racemic mixture. Similar results were obtained for SERS-l-MIPs and related l-enantiomers. Interestingly, all plots suggested a linear correlation, with coefficients of *R*^2^ *>* 0.9330. It is evident that the proposed IRM is solely responsible for a specific recognition reaction.

To verify the absolute enantiospecificity of the IRM, the responses of SERS-d-IPs and SERS-l-IPs to pure l-enantiomers and d-enantiomers, respectively, were studied. As depicted in Supplementary Fig. 5, although wrong enantiomers were nonspecifically bound on SERS-CIP, there was no difference between the responses of SERS-CIP to the wrong enantiomers and blank solutions. Thus, cysteamine inspection recognizes that there is no specific binding in the imprinted cavities of SERS-CIP. This is valid evidence that nonspecific recognition is curbed and absolute enantiomeric discrimination is realized.

For the specificity test asparagine, tryptophan, and glucose were chosen as target chiral models. Corresponding SERS-CIPs for asparagine, tryptophan, and glucose enantiomers were constructed. The specificity of each SERS-CIP based on the IRM was assessed by using a mixture of four l- and d-interfering enantiomers. The concentration of the target enantiomers were 1, 10, and 100 µg L^−1^, which were 100-fold lower than that of interfering enantiomers (100, 1000, 1 × 10^4^ µg L^−1^, respectively). As indicated in Supplementary Fig. 6, there is no apparent difference between the response to target enantiomers and that to the mixture of target enantiomers and interfering enantiomers. These results demonstrate that the developed IRM offers supreme selectivity. Such selectivity in addition to the spatial shape matching among the well-manufactured imprinted cavities and the target enantiomers stemmed from the ability of the IRM to curb nonspecific binding.

### Real-world application of SERS-CIP

Often, only the eutomer of medicinal drugs and pure enantiomeric (homochiral) chiral hierarchical structures such as amino acids and carbohydrates tend to be active in having their desired therapeutic, optical, and mechanical, properties, whilst the other enantiomers or enantiomeric mixtures can have lowered activity, be inactive, or biologically toxic^49^. The understanding of how homochirality can be reached from initially mixed heterochiral enantiomers is therefore imperative for many reasons. In this direction, scientists in chiral chemistry have devoted large endeavors to discriminating a wide variety of chiral compounds. Recognition of amino acids and carbohydrates as the most important bioactive chiral molecules in real samples is greatly significant in two main aspects: (i) chiral amino acids and carbohydrates are biomarkers of health and metabolic, renal, cardiovascular, cancer, and cerebrovascular diseases^50^. l-amino acids are used to express structural and functional proteins, while d-amino acids are non-protein amino acids used as intermediates in the production of peptide antibiotics and immunosuppressive drugs for the treatment of cancer and neurogenic diseases. (ii) Amino acids are ideal molecular indicators for the bioactivities of microorganisms and the degradation history of biological species and organic matter in the environmental matrices. For example, amino acids are important components of marine dissolved organic carbon pool and they are exerted into the seawater due to a variety of mechanisms, such as zooplankton excretion, direct release by phytoplankton via passive diffusion or active excretion, and bacterial exoenzyme production. d- and l-amino acids demonstrated different bioavailability. It is generally regarded that l-amino acids can be accumulated and used by microorganisms as carbon or nitrogen sources, while most d-type amino acids were inert. Thus, it is crucial to discriminate the concentrations of the enantiomers to elucidate their different contributions to marine carbon storage^51,52^. Therefore, the discrimination between right- and left-handed enantiomers of amino acids and carbohydrates, has received increasing concerns owing to its pivotal importance not only in biology, chemistry, pharmacology, and life sciences but also in healthcare^50^.

Often, chiral compounds, such as amino acids and carbohydrates have a low abundance in real samples and/or are accompanied by a high concentration of cross-contaminants. This is why the sensing performance of the corresponding sensors is severely affected when they are applied for real sample analysis^1,53^. Hence, for efficient analysis of real samples with complex matrices sample preparation is required, which commonly involves centrifugation, pH adjustment, etc. However, the implementation of sophisticated sample preparation, as is done in many conventional MIP-SERS platforms^54^, is not desired. Ideal MIP-SERS platforms should have good practical applicability and a real impact that works in actual samples.

**Fig. 8:**
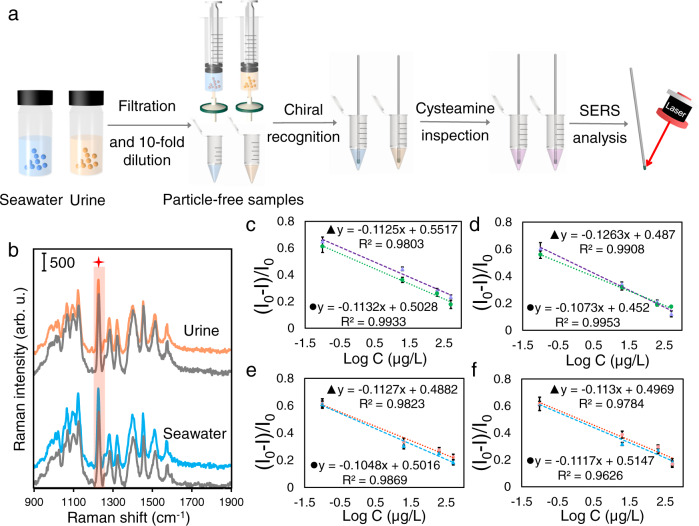
Real-world application of SERS-CIP. **a** Schematic illustration of real-sample analysis by the SERS-CIP based on IRM. **b** Raman spectra of DTTC on the SERS tag@capillary before (gray spectra) and after (blue and orange spectra) incubation in crude seawater and urine samples. Linear relationship between the relative SERS intensity ((*I*_0_−*I*)/*I*_0_) of **c**, **e** asparagine-SERS-CIP and **d**, **f** galactose-SERS-CIP and the logarithm of enantiomers concentration in the samples. *I*_0_ and *I* are the SERS intensities of DTTC before and after IRM, respectively. ▲ (purple and blue) and ● (green and pink) are related to d-enantiomers and l-enantiomers, respectively. **c**, **e** and **d**, **f** are related to seawater and urine samples, respectively. The error bars represent the standard deviation for four parallel experiments. Source data are provided as a Source Data file.

To appraise the practical applicability of the IRM, the SERS-CIPs were applied for the enentiodetection of asparagine and galactose in urine and seawater samples with simple dilution and filtration pretreatment (Fig. 8a). To verify the stability of the DTTC on the surface of Au NSs in the presence of matrix components, the SERS signal of SERS tag@capillary, which was incubated in urine and seawater was detected. As seen in Fig. 8b, the SERS intensity of DTTC remains unchanged suggesting no decomposition/degradation of DTTC occurred in both urine and seawater samples. In the next stage, the IRM was implemented on the SERS-CIPs, which were incubated with a series of urine and seawater samples spiked with a known chiral concentration. After cysteamine inspection and SERS measurements, the relative SERS intensity was plotted against the logarithm of the concentration of the target enantiomer in the spiked samples. The obtained plots obeyed a good linear relationship (*R*^2^ ≥ 0.9784), which is very close to the calibration curves (Fig. 8c–f), suggesting a negligible influence of the matrix and no blocking of the PDA binding sites by the matrix components was befallen. These results show the excellent anti-interference ability of the IRM. As a result, the original concentration of the target enantiomer from the unspiked real samples can be calculated in accordance with the matrix-matched calibration curve. Two different concentrations of target enantiomers, corresponding to the response range of the SERS-CIP, were separately spiked into the seawater and urine samples and considered as a criterion of accuracy. The SERS-CIP exhibited a response to the spiked target enantiomers via enhanced SERS intensity. The corresponding results are listed in Supplementary Table 2. Excellent recoveries in the range of 84.8–103.3% for l-asparagine, 82.8–96.5% for d-asparagine, 89.9–101.8% for l-galactose, and 88.3–108.4% for d-galactose with low standard deviations (SDs) are valid evidence to confirm the high practical applicability of the SERS-CIP for chiral recognition.

Another important aspect that should be concerned in appraising the real-world application of the SERS-CIP is reusability. Although after implementation of the IRM recovery of CIP can be easily performed, returning the SERS tag layer to its original background condition is challenging. Descriptions regarding the reusability requirements are provided in the Supporting Information. Therefore, the SERS-CIP is not reusable, and an individual SERS-CIP was used for each experimental run. However, it should be noted that by considering green, cost-effective, fast, facile, and scalable construction procedures, as well as satisfactory reproducibility of the chips prepared from different batches, one-time use of the developed SERS-CIP is not a remarkable disadvantage but an acceptable alternative for excellent applicability.

### Comparison with SERS-based chiral discrimination strategies

Creating the chiral anisotropy by a polarizable chiral platform following SERS detection^55^, label-free based on charge transfer^21^, and direct sensing^19^ are three commonly accepted mechanisms used for the identification of enantiomers by SERS. In these chiral SERS discrimination strategies producing asymmetric recognition sites on the nanostructure surface to provide local chiral environments for preferential adsorption of enantiomers is essential, which is face five main challenges: (i) Creating asymmetric colloidal nanostructures is difficult and sophisticated owing to the lack of easy control over the formation of chiral characteristics. (ii) The poor affinity of asymmetric colloidal nanostructures to most chiral compounds prevents the effective formation of analyte–surface interactions, which hampers efficient enantiorecognition and sensitive SERS detection. (iii) High susceptibility of these methodologies to matrix interferences, especially to matrix compounds, which possess intense Raman scattering. They also only work effectively for the discrimination of enantiomers of a chiral molecule, and there is a great challenge for enantiorecognition in mixtures containing different concentrated enantiomers. (iv) Insufficient enantiosensitivity due to the weak Raman scattering of chiral compounds. (v) In the aforementioned SERS-based strategies the whole chiral discrimination process from the platform construction to chiral recognition is laborious, time-consuming, and intricate, thus, requiring a highly expert operator to implement.

In contrast to current chiral SERS sensing, the IRM benefits from several advantages. First, all modification reactions, materials fabrication, and sensing operations involved in this protocol are very simple and conducted under mild conditions without complicated equipment, so that even untrained operators can easily construct SERS-CIP of good quality and perform analysis process. Second, the IRM is independent of Raman scattering of chiral molecules and has absolute enantiospecificity and high sensitivity, which are three key factors enabling discrimination of a wide variety of chiral molecules. Third, the IRM is easy-to-implement, fast, and the key steps of the procedure, from chiral recognition to Raman spectrum readout, require only ~11 min. Compared with techniques that take much longer (e.g. >12 h), the IRM has evidently higher throughput. The detailed step-by-step protocol of the IRM is appraised in the Supporting Information. Fourth, the IRM exhibited excellent real-world practical applicability, enabling absolute chiral recognition in the mixture of different enantiomers and complicated real matrices. For these reasons, compared with current SERS-based chiral discrimination strategies, the IRM has more extensive potential application in the chiral field.

In summary, the origin of nonspecific recognition of the wrong enantiomer in CISs that prevents absolute chiral discrimination is systematically identified by CD, isothermal titration calorimetry, and FT-IR. An IRM is proposed for curbing the nonspecific recognition of imprinted materials, and absolute chiral discrimination is realized. The key to the success of this mechanism in distinguishing specific recognition from nonspecific recognition is the scrutiny of all imprinted cavities of CIP by an inspector, which reflects their status to inspector-sensitive SERS substrates. The whole IRM-based chiral discrimination process from platform construction (involves green synthesis protocol without additional modification or post-fabrication), chiral recognition (comprises just dipping SERS-CIP into the solution under test), to SERS detection is very simple, without requiring complicated equipment, so that can be performed by less experienced operators. In contrast to current SERS-based chiral discrimination strategies, the IRM is label-free and independent of Raman scattering of chiral molecules, which not only assures the method sensitivity but also meets the requirements of versatility. To implement the IRM on the other imprinted systems for chiral discrimination of different target chiral compounds, a suitable inspector molecule can be selected among commercially available linear shape aminothiol molecules taking into account chiral imprinted cavities size. Analysis of racemic solutions and the mixture of enantiomers revealed that the IRM is completely exclusive toward specific recognition of imprinted materials, which is important in the accurate measurement of the enantiomeric purity. Importantly, real-world application of the IRM demonstrated the IRM is not susceptible to matrix interferences and SERS-CIP can be used for accurate chiral recognition in complex samples with high throughput. Therefore, the current work represents a critical extension of the concept of chiral recognition by imprinted materials, and the longstanding inherent defect of imprinting-based systems, namely, nonspecific binding, is overcome. Based on the absolute specificity, versatility, rapidity, and sensitivity of the IRM, we can foresee that the developed CIP has great promise in diverse applications, particularly specific recognition of chiral intermediates in biological and artificial systems, chiral catalysis, and chiral synthesis.

## Methods

### Reagents and materials

l-Arginine (98%), d-Arginine (98%), l-Asparagine (99%), d-Asparagine (99%), l-Histidine (99%), d-Histidine (99%), l-Tryptophan (99%), d-Tryptophan (98%), l-Glucose (98%), d-Glucose (≥99.5%), l-Galactose (99%), d-Galactose (99%), cysteamine (95%), d-Alanyl-d-Alanine (99%), l-Alanyl-l-Alanine (98%), L-Ribose (98%), D-Ribose (≥99%), L-Aspartic acid (99%), d-Aspartic acid (98%), 3-Aminopropane-1-thiol hydrochloride (3-ATH, 98%) and 6-aminohexane-1-thiol hydrochloride (6-ATH, 95%) were obtained from Shanghai Aladdin Bio-Chem Technology Co., Ltd. N-(2-hydroxyethyl) piperazine-N′-(2-ethanesulfonic acid) (HEPES, ≥99%) and dopamine hydrochloride pure powders (98%) were purchased from Beijing Solarbio Science & Technology Co., Ltd, and Sigma-Aldrich Chemical Co, respectively. 3.3’-Diethylthiatricarbocyanine iodide (DTTC, 99%) was supplied from Sigma-Aldrich. Chloroauric acid terahydrate (HAuCl_4_·4H_2_O), (3-Aminopropyl) trimethoxysilane (99%), RBS™ 25 solution, sulfuric acid (H_2_SO_4,_ 98.08%), hydrogen peroxide (H_2_O_2_, 30%), absolute ethanol (99.7%), acetic acid (CH_3_COOH, 99.5%), sodium hydrogen phosphate, and trizma hydrochloride (Tris–HCl, ≥99.9%) were obtained from Chengdu XiYa Chemical Technology Co., Ltd. All other reagents were of analytical grade and used as received. Glass capillaries with an inner diameter of 0.5 mm and a length of 100 mm were supplied from instrument factory of the west China medical university. Ultrapure water with specific resistance of 18.2 MΩ was prepared with Millipore, USA water purification system.

### Instruments

Scanning electron microscopy (SEM) images were recorded by an S-4800 field emission scanning electron microscope (Hitachi, Japan). Transmission electron microscopy (TEM) images were obtained on a JEM-1400 transmission electron microscope (JEOL, Japan). Atomic force microscopy (AFM) images were obtained on an atomic force microscope (Veeco, USA). Static water contact angles were measured using an OCA50 system (Dataphysics, Germany). Circular dichroism (CD) spectra were collected on the Chirascan spectropolarimeter. Isothermal titration calorimetry (ITC) was performed using a Nano calorimeter (Waters, TA Instruments, USA). FT-IR spectra were obtained from a Fourier transform infrared spectrometer (Nicolet IS10, Thermo Fisher). Zeta potential was detected on Malvern Zetasizer Nano-ZS90 (ZEN3590, UK). The UV−Vis absorption spectra were recorded on a Thermo Scientific NanoDrop 2000/2000C spectrophotometer. Fluorescence spectra were recorded on a HORIBA Scientific Fluoromax-4 spectrofluorometer.

### SERS measurement

The SERS spectra were collected on a DXR Raman microscope (Thermo Scientific, USA). The excitation light source was a He−Ne laser operating at *λ* = 780 nm, and the laser spot was focused on the platform through a ×10 objective lens. The excitation laser wavelength was selected by considering the absorption wavelength of DTTC. All spectra were measured with an exposure time of 2 s and unless otherwise specified, the Raman laser power was 30 mW. The baseline correction of Raman spectra was conducted using OMNIC for dispersive Raman 8.3.104 series software (Thermo Fisher Scientific Inc.).

### Synthesis of Au NSs

Au NSs were synthesized by a one-pot, seedless, surfactant-less, and green method. Briefly, 100 mM aqueous standard solution of HEPES was prepared with ultrapure water, and pH was adjusted to 7.5 at room temperature by adding 1 M NaOH solution. 2 mL of the above HEPES solution was mixed with 3 mL of ultrapure water, followed by the addition of 40 μL HAuCl_4_ solution with a concentration of 24.25 mM. The solution was maintained at room temperature without shaking, and its color was changed from light yellow to colorless, light gray, purple, and finally to blue, within 20 min.

### Construction of SERS-active glass capillaries

Sealed glass capillaries were submerged in RBS™ 25 solution under sonication at 80 °C for 20 min. Then, the capillaries were rinsed with ultrapure water repeatedly followed by immersion in Piranha solution and remained at room temperature for 4 h to clean and hydroxylate capillary surfaces. The capillaries again were rinsed with a high amount of ultrapure water and dried at 75 °C overnight. For amino functionalization, clean glass capillaries were vertically immersed in a 1% (v/v) ethanol solution of APTES in anhydrous ethanol at 70 °C for 6 h. After silanization, capillaries were sonicated and washed three times with ethanol to remove unreacted silane, and baked for 2 h at 100 °C in an air oven. At the next stage, the amino-modified glass capillaries were dipped into the Au NSs colloidal suspension to prepare Au NSs layer (this layer can be seen by the naked eye). Finally, glass substrates were washed with ultrapure water three times and were dried for 30 min at 30 °C. SERS-active glass capillaries were thus simply synthesized.

### Preparation of SERS tag

SERS-Active glass capillaries were dipped into the 10^−4^ M DTTC solution. Afterward, capillaries were rinsed with ultrapure water to remove excess DTTC molecules and dried at 30 °C. SERS tag was formed on the surface of glass capillaries and termed “SERS tag@capillary”.

### SERS-CIP construction based on mussel-inspired surface imprinting

In detail, 2.5 mg l- or d-enantiomer of amino acid and 10 mg dopamine hydrochloride were dissolved in 10 mM Tris–HCl buffer (pH 8.5). SERS tag@capillaries were vertically dipped in the above solution and incubated at room temperature for 3 h to complete the imprinting. Finally, template molecules were removed from the PDA network by washing SERS-CIP with a 0.5% (v/v) solution of acetic acid solution five times, and then thoroughly washed with ultrapure water to completely remove the residual acetic acid. The control SERS nonimprinted platforms (SERS-NIP) were constructed in the same manner as their corresponding SERS-CIP but without the addition of the template.

### Synthesis of chiral imprinted polydopamine particles (CIPPs) and nonimprinted polydopamine particles (NIPPs)

CIPPs were prepared according to self-polymerization of dopamine in the presence of l-Tryptophan or d-Tryptophan enantiomer, respectively. 0.15 g l-Tryptophan or d-Tryptophan and 0.8 g dopamine hydrochloride were dissolved in 10 mM Tris–HCl buffer (pH 8.5) and kept under stirring for 3 h to complete polymerization. Afterward, the particles were collected by centrifugation (15 min, 4500 × *g*) and washed several times with acetic acid 1% v/v and deionized water to completely remove template molecules. Finally, the obtained CIPPs were dried in a vacuum oven at 40 °C. The corresponding (NIPPs) were prepared with the same protocol except in the absence of Tryptophan enantiomers. The SEM images of l-IPPs and d-IPPs are illustrated in Supplementary Fig. 7.

### ITC analysis

Before measurement, each solution was degassed to remove air bubbles. The CIPPs or NIPPs (5 mg mL^−1^) dispersed in phosphate buffer (10 mM) and loaded in a 300 μL ITC cell at 25 °C. l-Tryptophan or d-Tryptophan (50 μL, 4.9 mM) in the same buffer was titrated into the cell (2.5 μL each time, except for the first injection).

### Absolute enantiospecific recognition of chiral compounds by SERS-CIP

The enantiospecific detection of chiral compounds was performed by three simple steps involving enantiomer recognition, inspector inspection, and SERS sensing. The tip of the SERS-CIP (recognition zone) was vertically dipped into a 100 µL enantiomer or chiral solution of amino acid or a real sample (urine, seawater) and incubated without shaking for 10 min. Then, the recognition zone of the SERS-CIP vertically dipped into a 100 µL cysteamine solution (0.5 M) or another inspector without shaking for 30 s (depending on the chiral template) followed by plunging into the ultrapure water (3 s) to stop further inspector diffusion. Finally, the SERS intensity of DTTC on the SERS-CIP was analyzed with the Raman microscope.

### Urine and seawater sample preparation

Urine samples were collected from female healthy donors on different days. Seawater samples were collected from the Yellow sea in Yantai coastal zone, China. All samples were stored at 4 °C before use. Because urine and seawater samples contain solid particles, they were simply filtered through a 0.22 μm filter and diluted 10-fold with deionized water prior to analysis. No additional sample preparation was performed for analyzing real samples.

### Reporting summary

Further information on research design is available in the Nature Research Reporting Summary linked to this article.

## Supplementary information

Supplementary Information

Reporting Summary

## Data Availability

The data that support the findings of this study are available in this article and it’s Supplementary Information. Source data are provided with this paper.

## Source data

Source Data
