# Supplementary material for: Chiral molecular imprinting-based SERS detection strategy for absolute enantiomeric discrimination
Source: Nat Commun. 2022 Sep 30;13:5757. doi: 10.1038/s41467-022-33448-w (PMC9525700; doi:10.1038/s41467-022-33448-w)
Supplement: Supplementary file 1 — Supplementary Information [file 41467_2022_33448_MOESM1_ESM.pdf]

Supplementary Information

Chiral Molecular Imprinting-based SERS Detection Strategy for Absolute Enantiomeric Discrimination

Maryam Arabi,[1] Abbas Ostovan,[1] Yunqing Wang,*[1,2] Rongchao Mei,[1] Longwen Fu,[1] Jinhua Li,[1] Xiaoyan Wang,[1,3] and Lingxin Chen*[1,2,4]

[1] CAS Key Laboratory of Coastal Environmental Processes and Ecological Remediation, Yantai Institute of Coastal Zone Research, Chinese Academy of Sciences, Yantai 264003, China

[2] Laboratory for Marine Biology and Biotechnology, Pilot National Laboratory for Marine Science and Technology, Qingdao 266237, China

[3] School of Pharmacy, Binzhou Medical University, Yantai 264003, China

[4] Center for Ocean Mega-Science, Chinese Academy of Sciences, Qingdao 266071, China

---

*Corresponding authors. Tel/Fax: +86 535 2109130.

E-mail addresses: yqwang@yic.ac.cn (Y. Wang); lxchen@yic.ac.cn (L. Chen).

Table of Contents

1. Supplementary Note 1: Study of intermolecular interactions by isothermal titration calorimetry (ITC)
2. Supplementary Note 2: Evaluation of the intrinsic nature of recognition by FT-IR
3. Supplementary Note 3: Optimization
4. Supplementary Note 4: Diffusion rate of inspector
5. Supplementary Note 5: Reusability of the SERS-CIP
6. Supplementary Note 6: Overview of the procedure
7. Supplementary Note 7: Characterization of substrates and platforms
8. Supplementary Note 8: Stability of SERS-CIP
9. Supplementary Note 9: Mechanism of chiral recognition by imprinted PDA
10. Supplementary Note 10: Method sensitivity comparison
11. Supplementary Figures
12. Supplementary Tables
13. Supplementary References

Supplementary Note 1: Study of intermolecular interactions by isothermal titration calorimetry (ITC)

To study intermolecular interactions, isothermal titration calorimetry (ITC) as one of the most reliable methods is used.[1, 2, 3] This method measures the heat change during titration and is able to afford the binding constants and thermodynamic parameters such as changes in free energy (ΔG), enthalpy (ΔH), and entropy (ΔS).[4, 5] Since the ITC instrument design especially its cells and stirring system prevents the analysis of capillary based platforms, CIPPs and NIPPs as substitutes of SERS-CIP and SERS-NIP, respectively, were analyzed. After background subtraction to account for the dilution effect, the enthalpy (ΔH) and binding constant (K_a) were obtained by fitting the titration curves to the ITC models. The K_d values were calculated from $1/K_a$ and $\Delta G = -RT \ln(K_a)$, where R is the gas constant. ΔS was calculated from $\Delta G = \Delta H - T\Delta S$. In Supplementary **Figure 8**, the top panel shows the raw calorimetric data. The area under each peak represents the amount of heat generated at each ejection and is plotted against the molar ratio of the CIPPs and NIPPs to the guest. The smooth solid line is the best fit of the experimental data to the sequential binding and independent binding site models. Binding parameters were auto-generated and present inset. All reactions released heat, which is favorable for binding. The L-IPPs and D-IPPs exhibited stronger affinities (K_a) for binding corresponding enantiomers (Supplementary **Figure 8a-b**) than that binding wrong enantiomers (Supplementary **Figure 8c-d**), indicating enhanced binding specificity stems from the spatial arrangement of the chiral imprinted cavities. Although NIPPs lack chiral imprinted cavities, they possess a remarkable binding affinity to L- and D-Tryptophan indicated nonspecific binding has a great contribution to chiral recognition (Supplementary **Figure 8e-f**).

It is mandatory to mention that the characteristic of chiral imprinted PDA layer in the SERS-CIP is entirely different from than that CIPPs in terms of morphology, thickness, and chiral imprinting efficiency. Therefore, it is expected the obtained thermodynamic parameters and binding constants for CIPPs and NIPPs are different from the actual values of SERS-CIP

and SERS-NIP. Indeed, the design and preparation of chiral imprinting materials with strong binding for the chiral model molecules is not the aim of the current work. Thus, imprinting variables, which eventually can improve binding constants and thermodynamic data were not optimized in the preparation of CIPPs and NIPPs. In this research, we developed a versatile recognition mechanism (inspector recognition mechanism, IRM) to curb nonspecific recognition of chiral imprinted systems and enabling absolute chiral discrimination. Most importantly, the IRM is not dependent on the binding strength and is enforceable on almost all CISs even on the systems with very weak binding constants for the chiral guests.

Supplementary Note 2: Evaluation of the intrinsic nature of recognition by FT-IR

The intrinsic nature of recognition (specific and/or nonspecific) between chiral imprinted and nonimprinted polydopamine (PDA) and a chiral guest was explored by FT-IR analysis. It is well-known that the intrinsic mechanism of chiral recognition by the imprinted polymers is derived from different noncovalent bindings between functional groups of polymer framework and target chiral molecules as well as the spatial arrangement of the chiral imprinted cavities.[6, 7, 8] Accordingly, regardless of binding strength, it is expected the nature of intermolecular interactions between enantiomers and SERS-CIP or CIPPs is approximately similar. Tryptophan was selected as a chiral model for FT-IR analysis because of possessing a strong and unique characteristic peak around 744 cm^{-1} (corresponding to the in-plane deformation of ring −H groups[9, 10]), which not exists in the PDA FT-IR spectra (Supplementary **Figure 9a**). This peak was tracked to investigate the intrinsic nature of recognition. FT-IR spectra of CIPPs after recognition of corresponding enantiomers, CIPPs after recognition of competitor enantiomers, and NIPPs after recognition of L- and D-enantiomers were recorded. If Tryptophan captured by any of the receptor, it is expected that the intended peak emerges in the relevant FT-IR spectra. A characteristic peak around 744 cm^{-1} emerged in the spectra of L-IPPs and D-IPPs after recognition of L-Tryptophan and D-Tryptophan, respectively, demonstrating recognition of Tryptophan by CIPPs (Supplementary **Figure 9b**). In this case, chiral recognition occurred via specific bonding (perfect placement of enantiomers into the chiral imprinted cavities). After the recognition reaction of competitor enantiomers by L-IPPs and D-IPPs, Tryptophan's desired peak observed as well, suggesting entity of nonspecific binding in chiral recognition (Supplementary **Figure 9c**). Nonspecific binding (nonfitting geometry) participates unavoidably in chiral recognition and stems from: (i) the interactions among functional groups of polymer framework and chiral molecules that lead to unwanted perching of wrong enantiomer into the imprinted cavities, and (ii) surface adsorption of chiral molecules. Both types of nonspecific binding could participate in chiral recognition when competitor enantiomers captured by CIPPs. Although, Tryptophan enantiomers nonspecifically captured by NIPPs, the characteristic peak around 744 cm^{-1} is too weak in NIPPs (Supplementary **Figure 9d**). This may be due to the low sensitivity of FT-IR. Since, NIPPs lack chiral imprinted cavities, only type (ii) nonspecific binding can contribute to the chiral recognition. Hence, it is expected the number of nonspecifically recognized Tryptophan molecules by NIPPs is lower than that CIPPs (after recognition of competitor enantiomers). Accordingly, captured Tryptophan molecules by NIPPs cannot be detected by FT-IR.

Supplementary Note 3: Optimization

Surface-assembly time of Au NSs

To attain intense and homogeneous SERS signals and subsequently the best SERS sensitivity, Au NSs should be immobilized on the surface of amino-modified glass capillaries with the maximum density. Accordingly, the incubation time of amino-modified glass capillaries in Au NSs solution within the range of 1-11 h was optimized. As shown in Supplementary **Figure 10**, passing immobilization time from 1 to 10 h SERS intensity increased until all amino active sites of capillary glass saturated by Au NSs and the signal became fixed. Thus, the immobilization time of 10 h was selected for all experiments.

Template/dopamine mass ratio

Different mass ratios of template/dopamine examined to attain a durable imprinted layer containing essential binding sites. To guarantee the conservation of the imprinted cavities after template removal, suitable cross-linked PDA should form. Using much dopamine quantity, a highly dense polymer with too high thickness is created. Consequently, template molecules deeply buried and hindered the mass transfer of the template. In contrast, applying a low dosage of dopamine produced flimsy thin PDA layer and therefore imprinted cavities can be easily deformed, resized and selectivity reduced. Besides, such a PDA layer cannot block cysteamine penetration and unfavorably degraded DTTC molecules. In other words, the specific enantiomer recognition reaction cannot be attributed to the signal decrease of DTTC. Imprinted cavities acted as chiral specific binding sites and the only passageway of cysteamine inspector to access SERS tag synchronously. The number of produced imprinted cavities can be regulated by the amount of used template molecules. Low template quantity produced scant imprinted cavities and these binding sites are fully occupied even by trace amounts of chiral molecules, so the SERS-CIP is unusable for chiral recognition at high concentration. On the other hand, if the template level exceeds a certain amount, too many imprinted cavities are created and the SERS-CHIP does not applicable for the chiral discrimination at trace level. Several mass ratios of template/dopamine including 1/1, 1/2, 1/3, 1/4, and 1/5 mg/mg were tested and among them the mass ratio of 1/4 mg/mg was optimal. This optimization was performed for all intended amino acids and the optimal mass ratio for all of them is the same with 1/4 mg/mg.

Imprinting time

PDA is a robust adherent polymer easily obtained by oxidation of dopamine at alkaline pH under air.[11, 12] PDA is mainly prepared by three commonly used approaches, including enzymatic oxidation, electropolymerization, and solution oxidation.[13] Among these methods, due to simple polymerization process without using complicated instruments, solution oxidation was used for the construction of SERS-CIP. Several key factors in the solution oxidation process, including solvent,[14] pH,[15] oxidants,[16] monomer concentration,[17] and polymerization time[18] have significant influence on PDA morphology, film thickness, and reaction rate. It has been frequently reported that by simply adjusting the polymerization time, the film thickness of PDA can be nicely tuned at the nanometer scale.[19, 20] Generally, porogen acts as pore forming agents in the polymerization process.[21, 22, 23] In detail, porogen molecules can become trapped in the matrix, creating pores after polymer drying. The size and total

pore volume are depending on several factors mainly including, the nature and level of the porogen, degree of drying and condensation of the polymer. In this work, to prepare chiral imprinted PDA layer on the SERS tag, the polymerization reaction occurred in Tris-HCl buffer (pH 8.5). The buffer chemical composition could serve as a possible way to efficiently modulate PDA growth.[14, 24] Slow and moderate reaction rate of PDA formation in Tris-HCl buffer stems from dopaminequinine, as an intermediate product, is a crucial control point in the pathway, which could be targeted by the nucleophiles in Tris-HCl buffer, thus hindering the layer growth.[24] Besides, in the SERS-CIP construction process, after chiral imprinting no drying was performed. Therefore, the generated PDA layer has the least possible porosity, which ensures the effective execution of the IRM.

To find sufficient density of polymer layer, the permeability of PDA to inspector during the imprinting process was studied. At the same time intervals of starting the imprinting, the capillary removed from the polymerization solution, rinsed with deionized water, dipped into the cysteamine solution and followed by SERS analysis (Supplementary **Figure 11**). At the beginning of the polymerization SERS tag layer has no coverage and inspector molecules can freely access to the SERS tag layer, degrade DTTC completely; thus, SERS signal is vanished. During the early hours of the polymerization, the accessibility of inspector to the SERS tag layer limited gradually due to continuous layer-by-layer deposition of PDA containing chiral template (1-2 h). Finally, after 3 h of imprinting the SERS signals of SERS-CIP before and after incubation in the inspector solution are equal demonstrating a polymer layer with sufficient density formed, which is impermeable to the inspector flow. After template removal, the SERS signal suppressed entirely upon inspector incubation, implying inspector molecules permeated through chiral imprinted cavities and degraded DTTC molecules. The SERS nonimprinted platform (SERS-NIP) lacks chiral imprinted cavities and SERS tag covered by a uniform dense PDA layer. Therefore, no specific binding site for chiral molecules and no pathway for inspector flux exist. Therefore, because of the formation of the PDA layer with sufficient density, the signal of SERS-NIP remained unchanged after the inspector inspection. It is necessary to mention, analyzing SERS-NIP reveals the size and volume pores of PDA layer are less than the amount that allow the inspector to access to the SERS tag. Consequently, imprinting time of 3 h was set for further experiments.

The influence of polymerization time on the growth of PDA layer within imprinting process was investigated by SEM (Supplementary **Figure 12**). As seen, by increasing the polymerization time PDA layer thickness increased gradually. Additionally, by comparing SEM images of SERS-CIP and SERS-NIP, it can be concluded that the imprinting has no obvious effect on the SEM morphology of the PDA shell proving the robustness of the PDA layer.[25]

Eluent solvent

After the imprinting process, enantiomer template molecules should remove from the PDA network to produce vacant specific cavities. The eluent strength of the solvent should not be low since template molecules cannot completely eliminate. A low number of binding sites and limited cysteamine pathways are attained. Besides, too high eluent strength can cause an alteration in the physical and chemical properties of the imprinted cavities of the PDA network and the stability of SERS tag. The alteration in the shape and size of the imprinted

cavities during template removal leads to different conformations from its original structure. Therefore, absolute enantiospecific recognition is not observed in the later re-binding attempt. Besides, in this condition, detachment of DTTC molecules from the surface of Au NSs is another problem. For amino acid template removal different elution solvents such as acetic acid 0.5 % v/v solution, acetic acid 1 % v/v solution, acetic acid 3 % v/v solution, acetic acid 1% v/v solution in methanol, and acetic acid 3% v/v solution in methanol were tested. The results indicated that acetic acid 0.5 % v/v solution can completely remove template molecules and possess no effect on SERS tag. Moreover, the number of washing cycles in the range of 1-7 time(s) was also investigated and 4 repeated elution times were chosen as the optimal level.

Inspector concentration

The concentration of cysteamine solution is the other parameters, which have a significant influence on the IRM. A low concentration of cysteamine cannot thoroughly inspect all the imprinted cavities as well as degrade DTTC dye. On the other hand, too high concentration can unfavorably degrade much amount of DTTC molecules even though only a low number of imprinted cavities are specificity unoccupied. To find the optimum concentration of cysteamine solution different concentrations, including 0.2, 0.5, 0.8 and 1 M were tested and experimental results revealed that 0.5 M cysteamine is the optimal value.

Time of IRM

Since the inspection of imprinted cavities by cysteamine flux occurs swiftly, the decisive step of the platform response time is related to enantiospecific recognition reaction. According to the time-dependent SERS responses of the SERS-CIP, which were obtained by incubations of SERS-CIP in the good enantiomer solution at different times in the range of 5-20 min; the optimum value of 10 min was achieved. Such a quick recognition time is related to the accessibility of the imprinted binding sites, which created into the thin PDA layer.

Supplementary Note 4: Diffusion rate of inspector

The diffusion rate is the time that takes for the inspector molecules to diffuse across the PDA network and reach the SERS tag. If the degradation reaction of DTTC by an inspector befalls fast, diffusion time is equal to the time from the incubation of platform in the inspector solution to complete signal suppression of the SERS tag. Therefore, by collecting inspector incubation time-dependent SERS spectra valuable information about the diffusion rate can be achieved. First, the degradation reaction time of DTTC by cysteamine on the Au NSs' surface was measured by tracking the SERS signal of SERS tag@capillary. As seen in Supplementary **Figure 13**, after incubation of SERS tag@capillary in cysteamine solution for one second, the SERS signal entirely vanished indicating the degradation reaction is completed within one second. Hence, the diffusion time of inspector within polymer film of SERS-CIP and SERS-NIP can be explained by suppressing its SERS signal. In other words, since degradation of DTTC by an inspector occurs quickly, the decisive step of the SERS signal change time after inspector incubation is related to the diffusion of inspector within the polymer layer. The diffusion time of inspector within imprinted PDA layer of SERS-CIP and nonimprinted PDA layer of SERS-NIP was studied. Supplementary **Figure 14** shows a

typical set of SERS spectra collected after inspector incubation at different times. For SERS-CIP, by passing time, SERS signals of DTTC gradually decreased, suggesting inspector motion within the polymer. Inspector molecules penetrate the polymer layer, access the SERS tag to some extent, and degrade a number of DTTC molecules. After 30 seconds, the SERS signal totally vanished due to the degradation of all DTTC molecules. Therefore, sufficient inspector molecules can diffuse into the all imprinted cavities of the PDA layer and inspect them after 30 seconds. SERS-NIP exhibited identical signals within 30 seconds, which demonstrated PDA network is impenetrable to inspector flow and cysteamine is an exclusive inspector for imprinted cavities.

Supplementary Note 5: Reusability of the SERS-CIP

After IRM, two events befall in chiral imprinted PDA and SERS tag layers. First, enantiomer molecules are located into the imprinted cavities of PDA. Second, chemical degradation of DTTC molecules occurs on the surface of Au NSs after cysteamine inspection. Reusability is the ability of a platform to recover or return to its original background/baseline condition, after recognition reaction. For this aim, three processes are essential to recover the proposed SERS-CIP, as follows: i) removing captured enantiomer molecules from imprinted cavities of the PDA layer; ii) removing degradation products of DTTC on the surface of Au NSs; iii) attaching fresh DTTC molecules to Au NSs. i) and ii) cases can be easily performed by dipping the capillary platform into the suitable rinsing solvent. However, the last one is challenging. The surface of Au NSs is thoroughly covered by the imprinted PDA layer and the only pathway to access the Au NSs surface is the imprinted cavities of PDA. Hence, to make DTTC molecules reach the Au NSs surface and form the SERS tag, DTTC molecules must penetrate through imprinted cavities of PDA. This phenomenon cannot befall effectively, since the size of chiral imprinted cavities is much smaller than DTTC molecular size. Therefore, spatial hindrance limits the accessibility of DTTC molecules to Au NSs. In addition, if the capillary platform incubates in DTTC solution with the aim of reusability, some DTTC molecules remain on the surface of the PDA layer and generate a false signal via shell-isolated nanoparticle-enhanced Raman spectroscopy (SHINERS) effect.[26] Such a signal could diminish after cysteamine inspection without any relation to specific enantiorecognition and lead to analytical error. On the other hand, remaining DTTC molecules on the surface of PDA may lead to spatial hindrance and interference with chiral recognition by the imprinted cavities. As a result, we have not obsessiveness to gain reusable SERS-CIP, but we have used an individual SERS-CIP for each experimental run. It should be noted that although the proposed SERS-CIP is not reusable, by considering green, cost-effective, facile, and scalable construction procedure, as well as excellent practical applicability, we are convinced that the platform is affordable, ideal and emerging for utilizing in many important applications, particularly in chiral recognition and discrimination.

Supplementary Note 6: Overview of the procedure

The detailed step-by-step protocol of the IRM is appraised in terms of requiring time, chemical consumption, and expertise of the operator. The general procedure of IRM is schematically illustrated in Supplementary **Figure 15** and consists of four main stages:

i) Platform construction is the most time-consuming step but uncomplicated. Most of the required time is dedicated to platform drying. To fabricate SERS tag on the surface of glass capillary three main steps are required: synthesis of Au NSs, amino functionalization of the glass capillary, and immobilization of DTTC on the surface of Au NSs. Au NSs are synthesized by a one-pot, seedless, surfactant-less, and green method with the least amount of reagents (only HEPES buffer, $HAuCl_4$, and deionized water) within only 20 min. Amino functionalization of the glass capillary is performed under mild conditions by using safe reagents (APTES and ethanol). Only by dipping SERS active substrate in DTTC solution and incubation for several seconds, SERS tag@capillary is established. In the design of the chiral imprinted layer, mussel-inspired surface imprinting is used as a sustainable, facile, and scalable strategy.[23] Solution oxidation of dopamine in the presence of the target enantiomer can be easily performed in aqueous media and at room temperature. The polymerization and template removal processes are only taken less than five hours. All modification reactions and materials fabrication involved in this protocol are very simple and conducted under mild conditions without complicated equipment so that even untrained operators can easily construct SERS-CIP of good quality. Evidently, this strategy can be preponderant in commercial manufacture and has great potential for scale-up. ii) Chiral recognition is taken place by just vertically dipping SERS-CIP into the solution under test and incubating for 10 min without shaking. iii) Scrutiny of the imprinted cavities' status is carried out by simply plunging SERS-CIP into the inspector solution and remaining for 30 seconds. iv) Finally, the SERS intensity of the SERS-CIP is analyzed by the Raman instrument. Some knowledge of Raman spectrometry is essential to accomplish the processing of signal acquisition, characteristic peak analysis and data interpretation.

As seen, the IRM is fast, and the key steps of the procedure, from chiral recognition to Raman spectrum readout, require only ~11 min. Compared with techniques that take much longer (e.g. >12 h[27, 28]), the IRM has evidently higher throughput and enables absolute chiral discrimination of wide varieties of chiral compounds.

Supplementary Note 7: Characterization of substrates and platforms

The inspector-sensitive SERS tag layer renders the sensitivity of the platform as well as translating the information of the inspector about binding status of the chiral imprinted cavities. SERS tag is created by attaching intrinsically strong Raman scattering molecules (called Raman reporters) to the surface of plasmon-resonant metallic NPs. Metal nanosubstrate act as structural scaffold and a Raman signal amplifier for the engineering of SERS tags. Generally, their size distribution, geometry, chemical composition, and surface chemistry can influence the Raman enhancement ability. In the current work, the combination of Au NSs and 3.3-Diethylthiatricarbocyanine iodide (DTTC) Raman reporter was selected to produce SERS tag. Au NSs possess strong SERS enhancement ability and DTTC has a large Raman scattering cross-section. This combination is helpful to gain a sensitive surface-enhanced resonant Raman scattering (SERRS) effect under a 780 nm laser irradiation. It has been demonstrated that the localized surface plasmon resonances of branched Au NPs, such as Au NSs, can be tuned by variation of their aspect ratio,[29] while the high electromagnetic field enhancements at their sharp tips render Au NSs superior Raman-enhancing substrates as compared to spherical Au NPs.[30] In addition, the increased surface area relative to that of a

sphere of equivalent size allows more Raman reporter molecules to be attached on multibranched NPs than on smooth-shaped NPs.[31] From the TEM image (**Fig. 3c**), Au NSs are a mixture of different-shaped particles, including spheres (much lower abundance) and branched particles (high abundance). The branched particles have star-shaped morphology (spherical core structures with protruding sharp tips, from one to eight tips), and these tips can act as outstanding "hot spots".[32]

For the construction of a SERS-based platforms, paper,[33] ITO glass slide,[34] wafer,[35] glass needle,[19] acupuncture needle,[36] and glass capillary[37] have been used as a substrate. Compared with prevalent substrates glass capillaries benefits from impressive advantages of: i) Glass capillaries with identical diameter are very cheap and commercially available everywhere. ii) Because of the smoothness of the glass surface, SERS tag uniformly immobilized on the glass and provides reproducible and homogenous SERS signal within a platform and inter-platforms. The surface of the glass was characterized by SEM and cross-SEM and corresponding images illustrate in Supplementary **Figure 16**. iii) Scalable capillary platform production can be easily achieved because of the small dimension and ease of manipulation. Tens of platforms can be simply constructed in one batch (Supplementary **Figure 17**). Moreover, a small amount of chemicals and reagents for platform construction and a very low volume of chiral solution (100 µL) for chiral discrimination/recognition are required, which greatly reduce the cost of analysis. iv) This novel platform geometry is versatile and robust for in-situ chiral recognition/discrimination. v) Implementation of chiral recognition/discrimination by the capillary platform is very simple and involves just dipping the capillary platform in the chiral and inspector solutions, respectively, followed by SERS detection. So recognition/discrimination can perform by an untrained operator.

SERS-CIP construction strategy is versatile and the size, geometry, and material type of substrate have not remarkable influence. Regardless of substrate type, the SERS-CIP consisted of two distinct layers, inner inspector-sensitive SERS tag and outer chiral imprinted polydopamine. The mechanism of Au NSs immobilization on the substrate is an electrostatic interaction. Hence, Au NSs can be effectively linked to any positively charged substrates.[38] PDA can be strongly immobilized on organic and inorganic surfaces through covalent and noncovalent bonds. Accordingly, the mussel-inspired chiral imprinted layer can be effectively immobilized on a wide variety of substrate materials in one step. Therefore, both Au NSs immobilization and mussel-inspired chiral imprinting can be realized on the various supporting substrates. Consequently, according to the intended application of SERS-CIP, the yield of SERS-CIP can be improved by using other substrates such as glass substrate with the larger size or even designing SERS-CIP in the form of colloidal particles.

SEM image of SERS-active glass capillary (Supplementary **Figure 18**) reveals Au NSs evenly anchored on the glass capillary surface. A high density of Au NSs and negligible degree of aggregation cause the generated SERS signals to become intense and homogeneous, which is the prevailing benefit of the electrostatically assisted surface-assembly method. It should be mentioned, little aggregation probably arises from the high quantity of Au NSs in solution before self-assembly and the polarization of Au NSs stimulated by the positively charged amino group of functionalized-glass.[39, 40]

To appraise the uniformity and reproducibility of the SERS-CIP, the SERS intensity of fifty random spots of six parallel SERS-CIP, which were constructed in six different batches,

was measured, and the results are displayed in Supplementary **Figure 19**. Based on statistical analysis, the relative standard deviations (RSDs) for six SERS-CIP were not higher than 5.7%, and this low RSDs demonstrate that the platform-to-platform variation in SERS intensity is very low. Such a low variation of SERS intensity arises from the uniformity of the SERS-CIP surface. Three key factors are simultaneously involved in the reproducibility of the SERS signals: 1) glass capillary has a very smooth surface, which is very beneficial for uniform SERS tag immobilization.[41] 2) The electrostatically assisted APTES-functionalized surface-assembly method was used for the fabrication of SERS active substrates. The dominant merit of this method is that a very uniform substrate with a very homogeneous SERS signal can be fabricated.[42] 3) Mussel-inspired surface imprinting was chosen for the construction of chiral imprinted layer. Solution oxidation of dopamine to form PDA is a facile polymerization approach; the PDA layer with a proper thickness and high reproducibility can be easily formed.[19]

The bare capillary glass, SERS-active glass (capillary glass@Au NSs), and SERS-CIP characterized by different instrumental techniques including energy dispersive X-Ray (EDX), cross-sectional SEM, atomic force microscopy (AFM), and contact angle. From cross-sectional SEM images (Supplementary **Figure 20a**), the capillary glass substrate has a very smooth surface. In SERS-active glass, a thin layer of Au NSs covered glass surface and caused the surface to be uneven (Supplementary **Figure 20b**). After polymerization, imprinted PDA layer buried Au NSs, and monotonously covered capillary@Au NSs substrate (Supplementary **Figure 20c and d**).

EDX analysis was performed to study the elemental composition of the samples. By comparison EDX spectra of bare glass capillary and capillary@Au NSs substrate, it can conclude Au NSs successfully anchored on the glass surface with a weight percentage of 4.95, since Au peaks emerged in the spectra of capillary@Au NSs (Supplementary **Figure 20a** and **b**). Both SERS-L-MIP and SERS-D-MIP possess a high content of O and C elements, which is related to the coated PDA layer (Supplementary **Figure 20c** and **d**). In addition, the weight percentage of all elements in SERS-L-MIP and SERS-D-MIP are very similar, indicating the reproducibility of the construction method. From EDX mapping, Au element uniformly distributed on the glass capillary without accumulation. Elemental mapping images of SERS-L-MIP and SERS-D-MIP show C and N that make up chiral imprinted polymers are distributed uniformly throughout the samples.

AFM further used to assess the materials' surface. Supplementary **Figure 21a** displays, Au NSs uniformly linked on the glass capillary and supplied an uneven surface. After the imprinting process, due to the homogeneous coating of the PDA layer and burial of Au NSs, the surface of both SERS-L-MIP, and SERS-D-MIP became relatively smoother (Supplementary **Figure 21b** and **c**). As expected, the surface characteristics of SERS-L-MIP and SERS-D-MIP are approximately identical.

The hydrophilicity of all substrates and platform was tracked by surface water contact angle (CA) measurement. As displayed in Supplementary **Figure 22**, the CA degrees for the bare glass capillary, amino-functionalized glass capillary, glass capillary@Au NSs, SERS tag@capillary, SERS-L-MIP, and SERS-D-MIP were 61°, 72°, 43°, 55°, 39°, and 34°, respectively. Immobilization of Au NSs on the glass capillary has little effect on the hydrophilicity. SERS tag is more hydrophobic substrate compared with glass capillary@Au

NSs because of the intrinsic hydrophobicity of DTTC molecules attached to the Au NSs surface. Compared with the SERS tag substrate, SERS-CIP has more hydrophilic surface indicated a uniform coating of PDA on the platform.

Supplementary Note 8: Stability of SERS-CIP

Stability of SERS-CIPs within long-term storage (35 days) in vacuum and ambient conditions was examined. In this regards, SERS signal of SERS-CIP at the same time intervals (7 days), was measured (Supplementary **Figure 23**). The reason for the storage of SERS-CIP in vacuum is to delay the oxidation process of materials. The signal of SERS-CIP, which kept in a vacuum remained almost unchanged after 35 days. It demonstrates both SERS tag and chiral imprinted PDA layers are stable in vacuum. The stability of Au NSs probably stems from Good's buffer, N-(2-hydroxyethyl) piperazine-N′-(2-ethanesulfonic acid) (HEPES), which act as surface stabilizing agent.[43, 44] However, the stability of SERS-CIP retained in ambient conditions is not as good as ones stored in vacuum since SERS signal of SERS-CIP decreased by passing time. This may be attributed to etching and/or restructuring of Au NSs into spherical nanoparticles by iodide ion of DTTC.[45, 46] To further investigate stability of materials, SERS-active glass capillary and SERS-CIP after 35 days storage in vacuum and ambient conditions characterized by SEM and the images presented in Supplementary **Figure 24**.

Supplementary Note 9: Mechanism of chiral recognition by imprinted PDA

It is well-known that functionality and spatial geometric shape of imprinted cavities could contribute for capturing target analyte by the imprinted materials.[6, 8, 47] In order to assess the recognition mechanism of chiral molecules by the imprinted PDA, the influence of pH in the range of 5.0-9.0 was examined. As mentioned above, PDA contains amine and phenolic hydroxyl groups, which makes it potentially ampholytic or zwitterionic. Therefore, the surface charge of PDA changed by pH variation. The surface charge of PDA and chiral models at different pH was measured by the zeta potential and the results presented in Supplementary **Table 3**. Although PDA and chiral models in all tested pH acquire different surface charges, the results of pH experiments demonstrated that pH has a negligible effect on the chiral recognition performance of SERS-CIP (Supplementary **Figure 25**). Accordingly, it can conclude that chiral recognition reaction could not occur based on the electrostatic affinity. The dominant recognition mechanism of chiral molecules by imprinted PDA layer may be the shape and spatial arrangement of imprinted cavities (shape selective). However, by considering the noncovalent functionalities of PDA and functional groups of chiral molecules, the participation of hydrogen bonding and π-π stacking interaction (for a chiral model that contains aromatic ring, Tryptophan) in chiral recognition can be expected.

Supplementary Note 10: Method sensitivity comparison

Various types of receptors with different recognition mechanisms have been developed for detection of chiral molecules. Since detection sensitivity of each method is extremely depends on the molecular properties of target chiral molecule, we choose common chiral targets, Tryptophan and Histidine, as chiral models for method comparison. The mechanism and sensitivity of different detection schemes including fluorescence, SERS, electrochemical,

photoelectrochemical, and CD for enantio recognition of Tryptophan and Histidine as chiral models were compared and presented in Supplementary **Table 1**.[48, 49, 50, 51, 52, 53, 54, 55, 56, 57, 58] Obviously, chiral detection based on IRM exhibited the highest sensitivity and the widest response range in comparison with the reported methods. More importantly, through IRM in addition to impressive improving sensitivity, nonspecific binding of CISs can be curbed and enables absolute chiral discrimination. Therefore, the long-term barrier of chiral imprinting technology is overcome.

Supplementary Figures

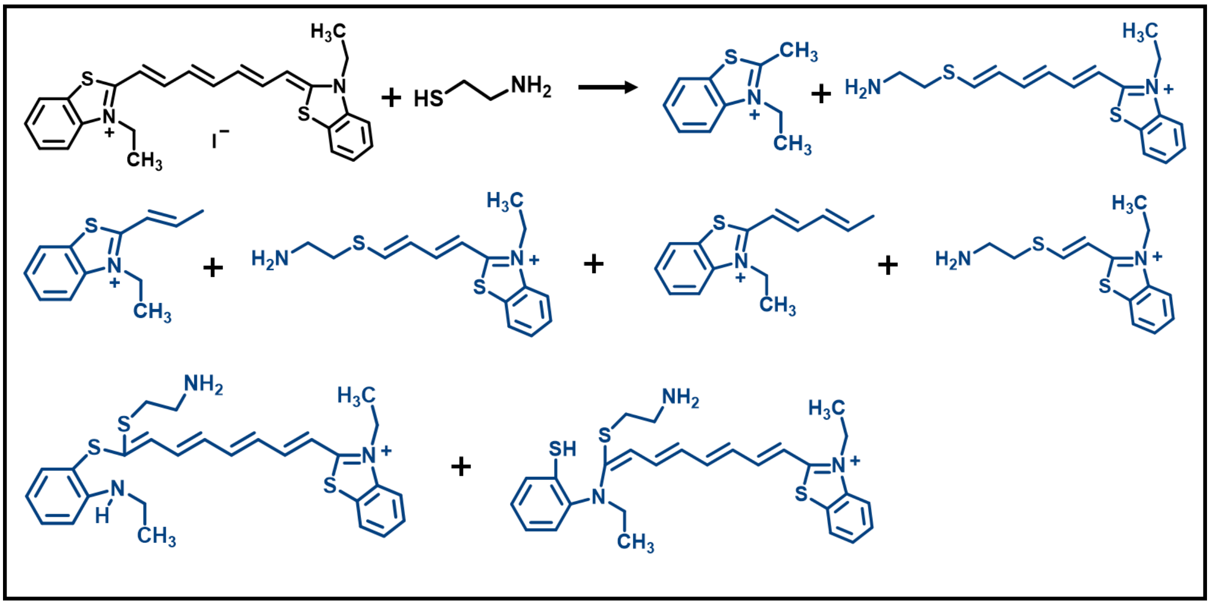

Supplementary Figure 1. **Chemical degradation of DTTC.** Degradation reaction of DTTC by cysteamine.

Mechanism I:

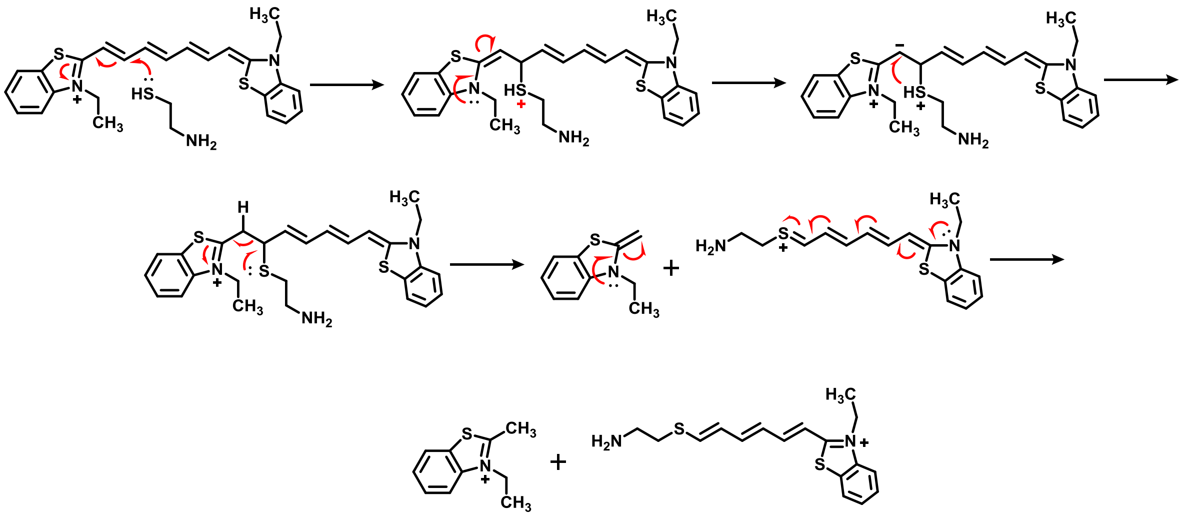

Product 1 **Product 2**

Mechanism II:

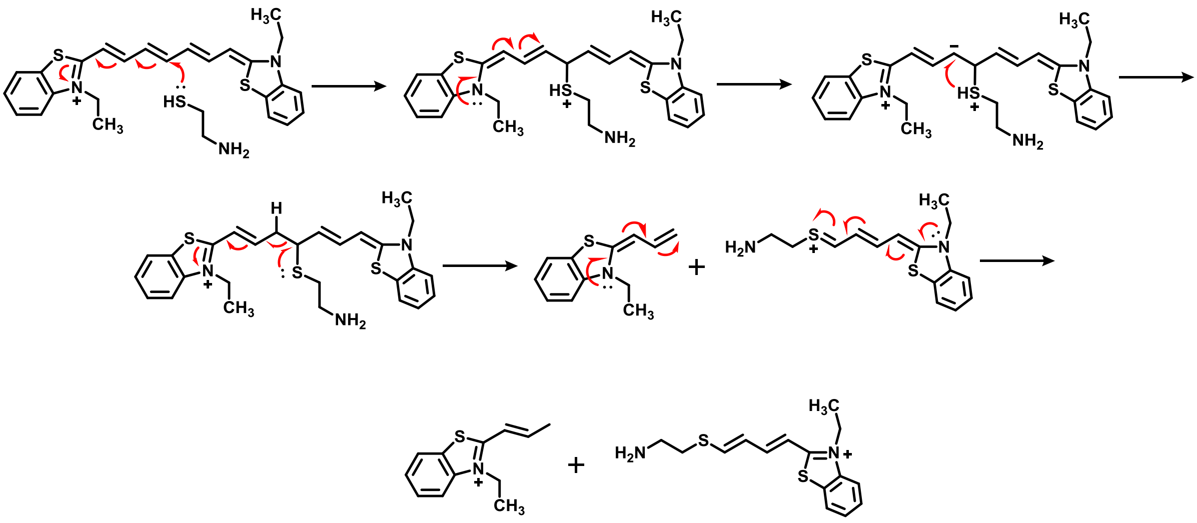

Product 3 Product 4

Mechanism III:

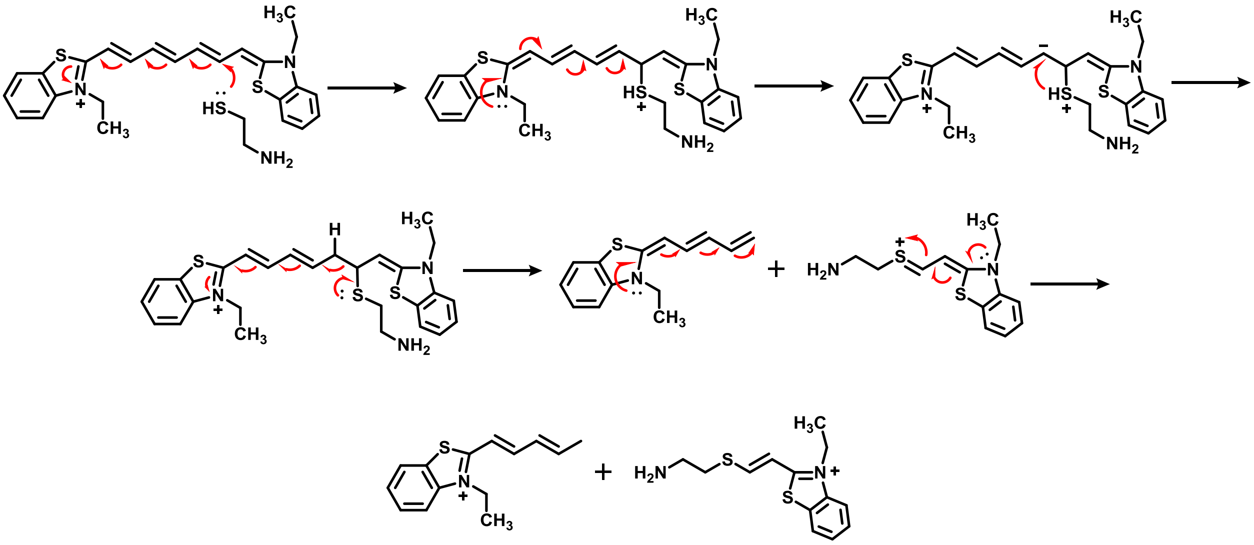

Product 5 Product 6

Mechanism IV:

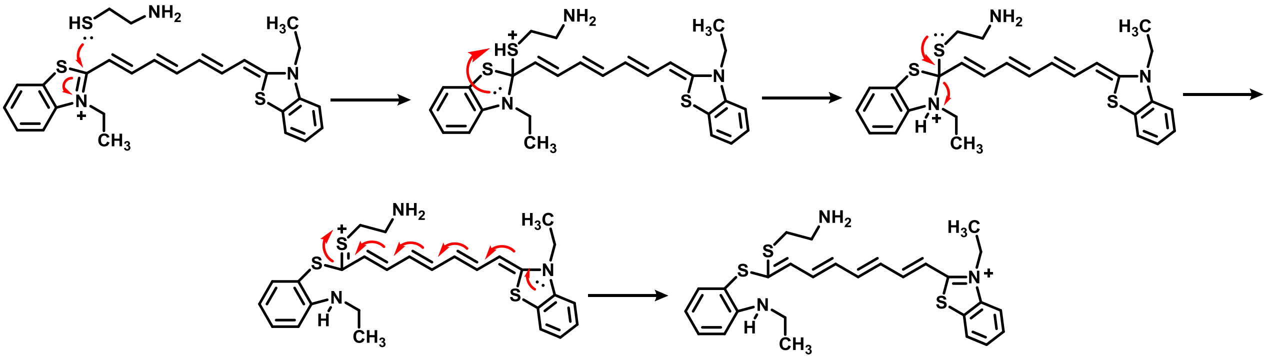

Product 7

Mechanism V:

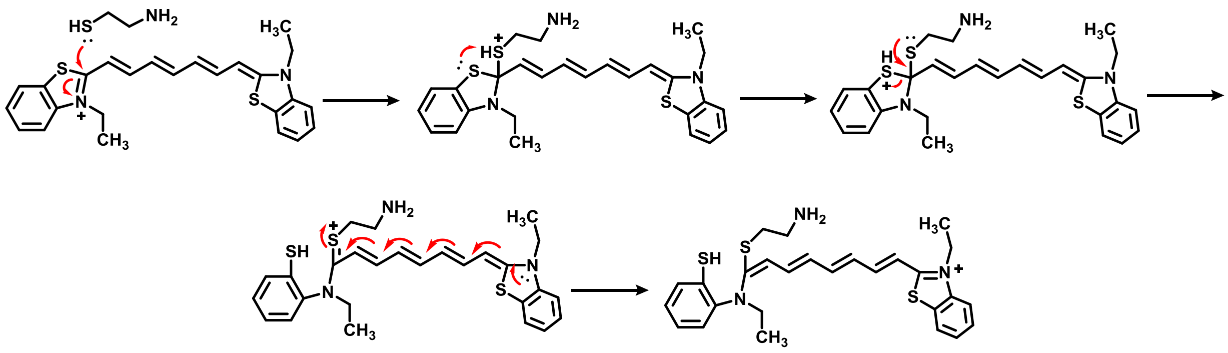

Product 8

Supplementary Figure 2. **Degradation mechanism.** Possible degradation mechanisms of DTTC by cysteamine.

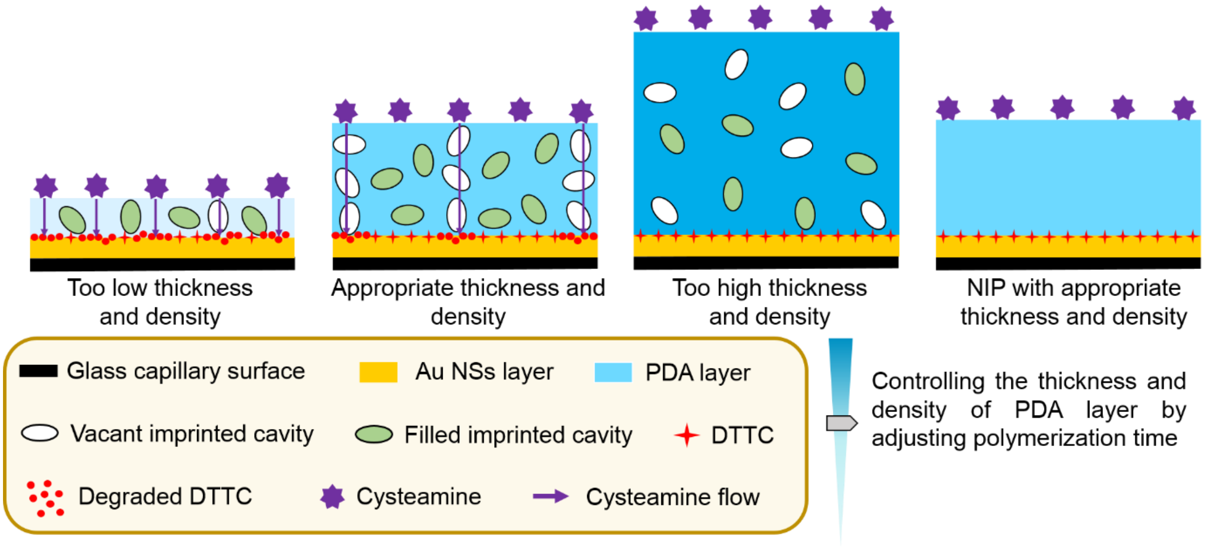

Supplementary Figure 3. **PDA layer thickness and density.** Influence of imprinted PDA thickness and density on the IRM.

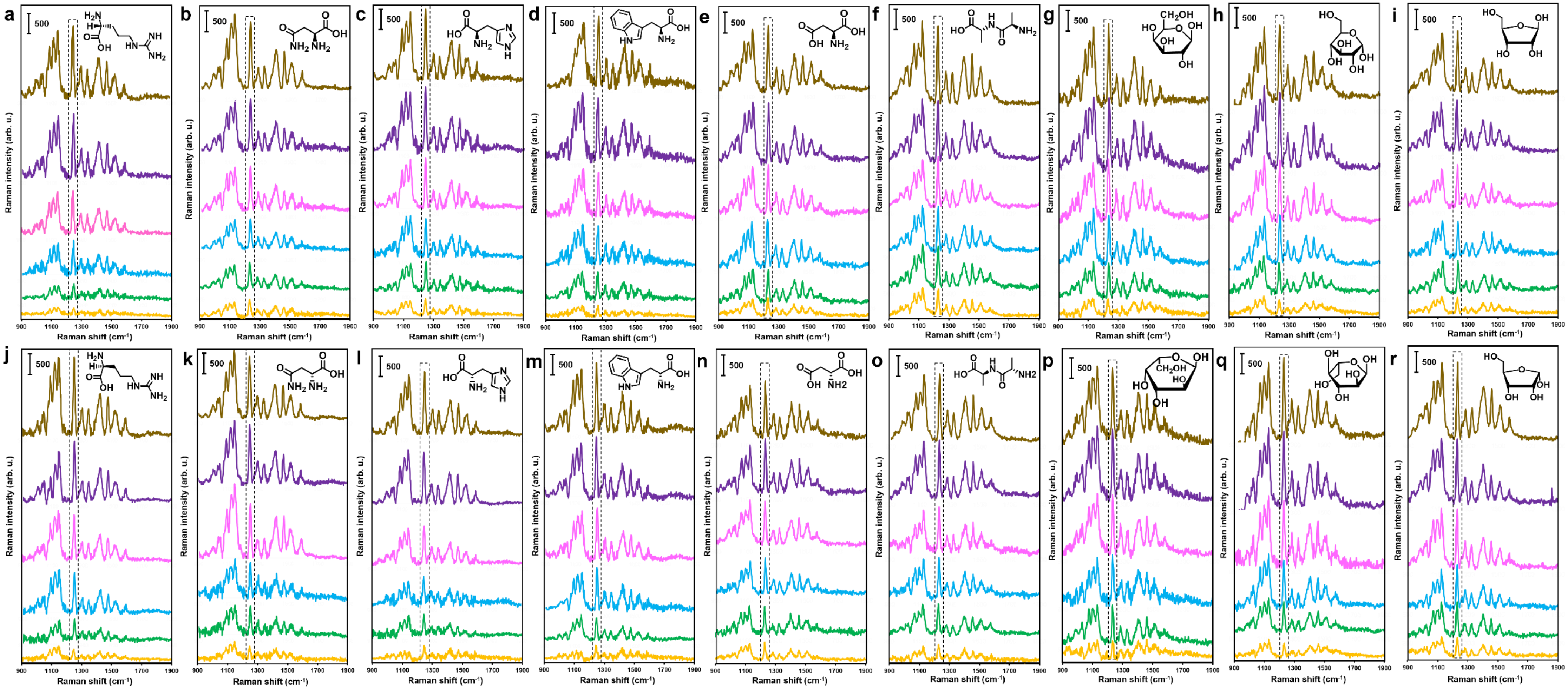

Supplementary Figure 4. **SERS spectra.** SERS spectra of DTTC on the **a** D-Arginine, **b** D-asparagine, **c** D-histidine, **d** D-tryptophan, **e** D-aspartic acid, **f** D-alanyl-D-alanine, **g** D-galactose, **h** D-glucose, **i** D-ribose, **j** L-arginine, **k** L-asparagine, **l** L-histidine, **m** L-tryptophan, **n** L-aspartic acid, **o** L-alanyl-L-alanine, **p** L-galactose, **q** L-glucose, and **r** L-ribose platform related to the calibration plots. Orange, green, blue, pink, purple, and brown colors demonstrate 0.001, 0.01, 1, 10, 100, 1000 μg L^{-1} concentrations, respectively. Source data are provided as a Source Data file.

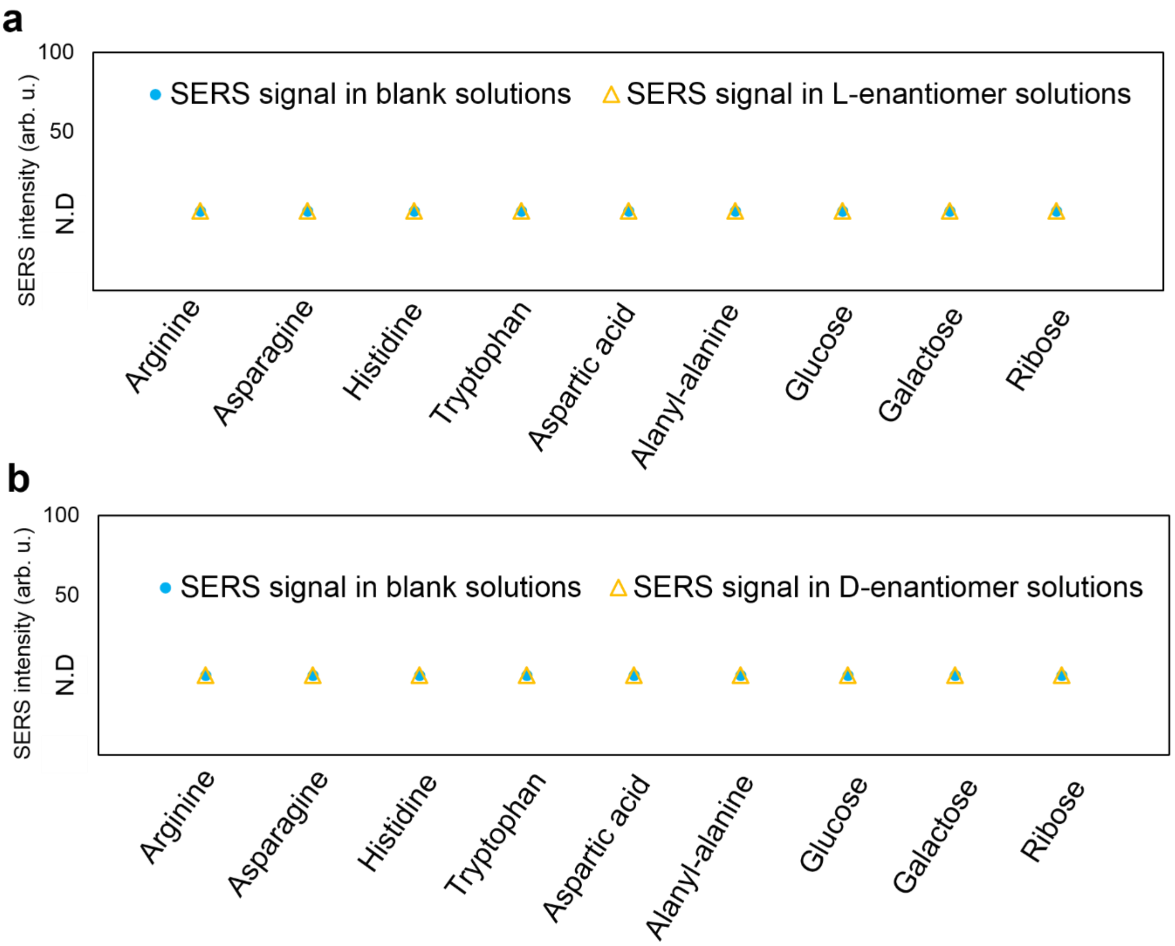

Supplementary Figure 5. **Enantiospecificity of the IRM. a** Response of SERS-D-IP to blank and L-enantiomer solutions. **b** Response of SERS-L-IP to blank and D-enantiomer solutions. N.D. = Not detected. Source data are provided as a Source Data file.

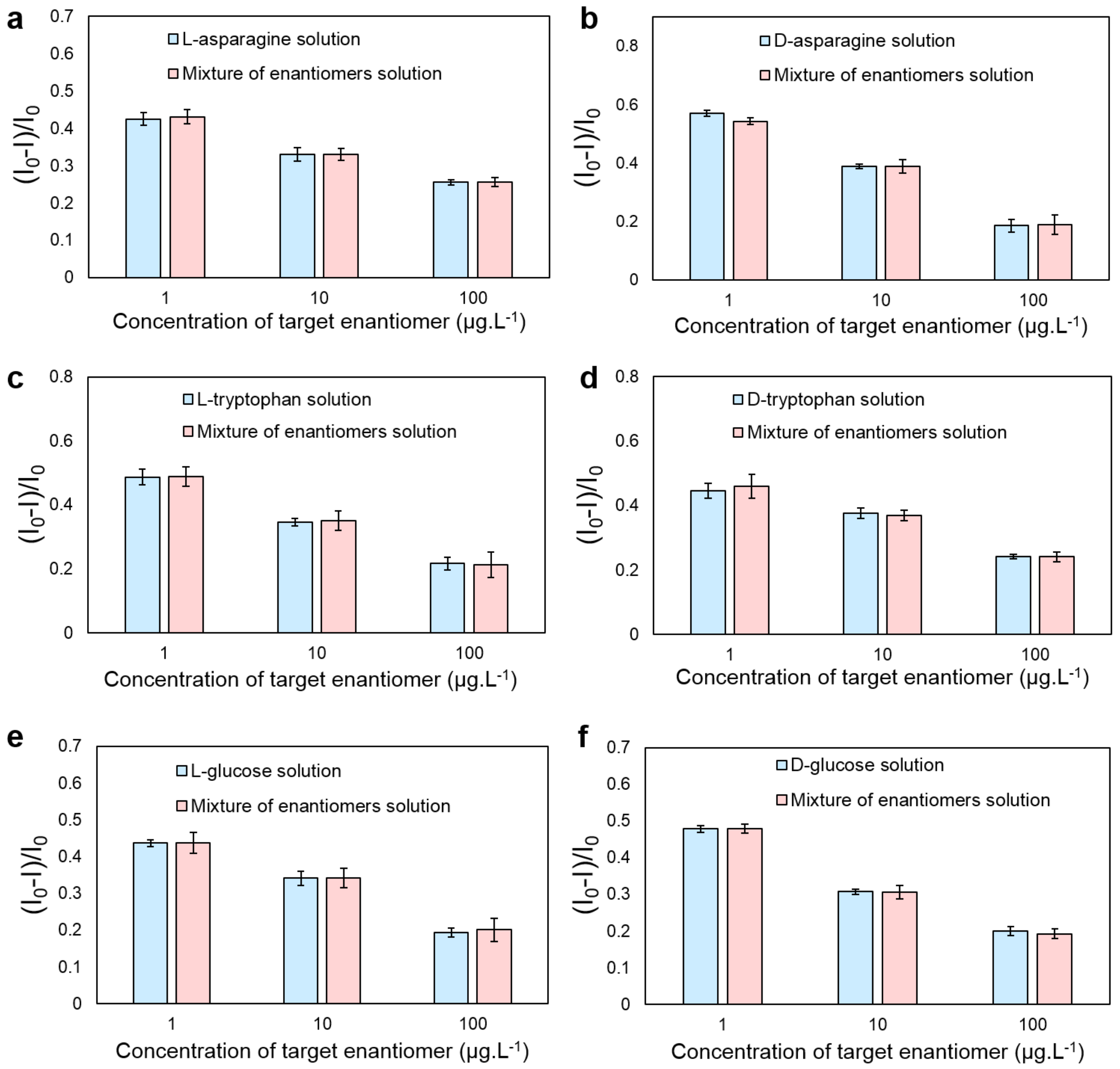

Supplementary Figure 6. **Specificity test.** **a** Response of L-asparagine-SERS-IP to L-asparagine and the mixture of L-asparagine, L-histidine, L-aspartic acid, d-histidine, and L-arginine solutions. **b** Response of D-asparagine-SERS-IP to D-asparagine and the mixture of D-asparagine, D-asparagine, D-histidine, D-aspartic acid, and L-histidine solutions. **c** Response of L-tryptophan-SERS-IP to L-tryptophan and the mixture of L-tryptophan, L-asparagine, L-arginine, L-histidine, and D-histidine solutions. **d** Response of D-tryptophan-SERS-IP to D-tryptophan and the mixture of D-tryptophan, D-histidine, D-arginine, D-asparagine, and L-histidine solutions. **e** Response of L-glucose-SERS-IP to L-glucose and the mixture of L-glucose, L-ribose, L-galactose, L-histidine, and D-ribose solutions. **f** Response of D-glucose-SERS-IP to D-glucose and the mixture of D-glucose, D-ribose, D-tryptophan, D-galactose, and L-ribose solutions. Error bars represent the standard deviations (n = 3). Source data are provided as a Source Data file.

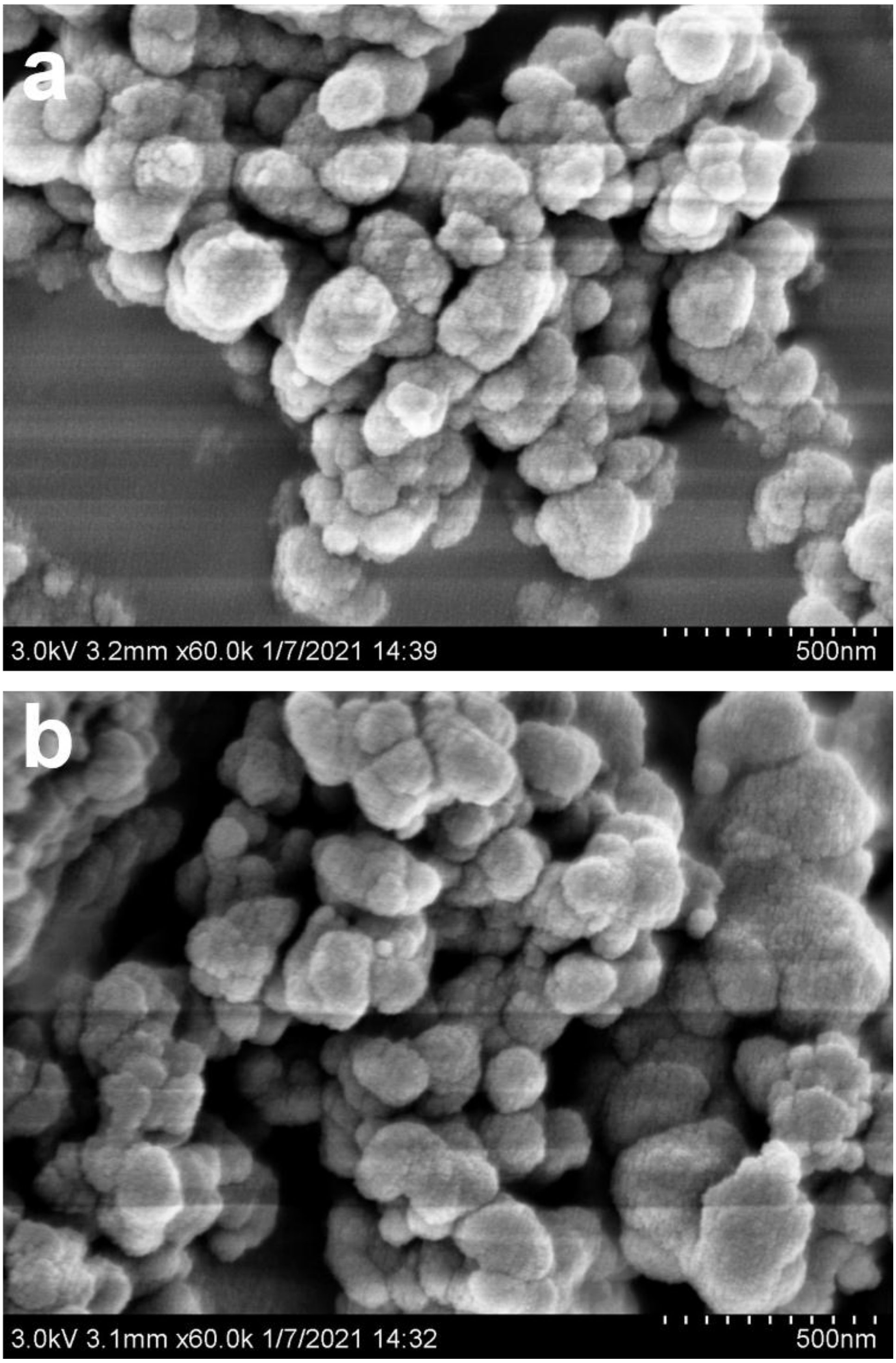

Supplementary Figure 7. **Morphology of CIPPs.** SEM images of **a** L-IPPs and **b** D-IPPs.

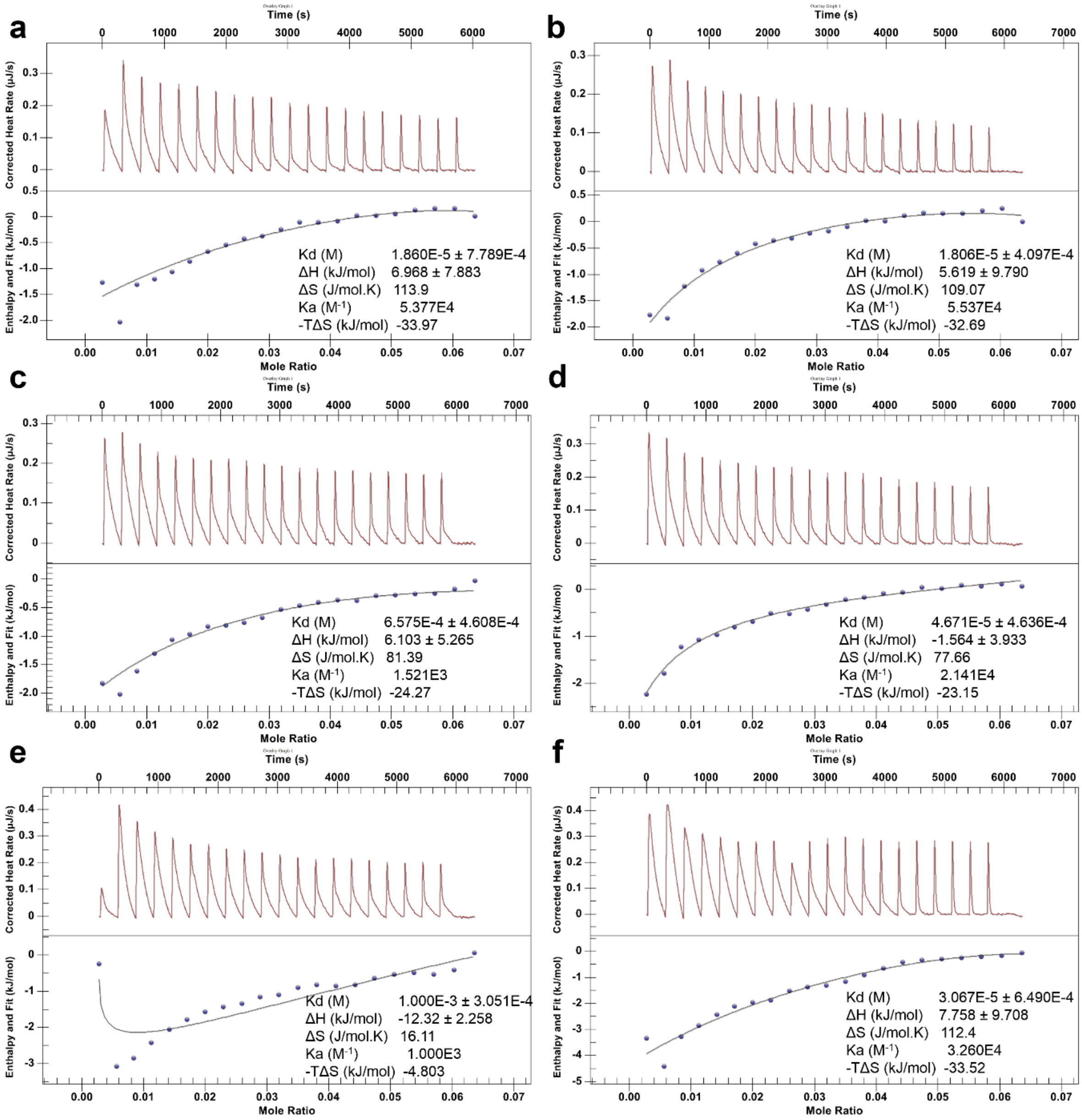

Supplementary Figure 8. **Binding constants and thermodynamic data.** ITC titration curves and related thermodynamic data obtained at 25 ℃ for the titration of **a** D-IPPs with D-Tryptophan, **b** L-IPPs with L-Tryptophan, **c** D-IPPs with L-Tryptophan, **d** L-IPPs with D-Tryptophan, **e** NIPPs with D-Tryptophan, and **f** NIPPs with L-Tryptophan.

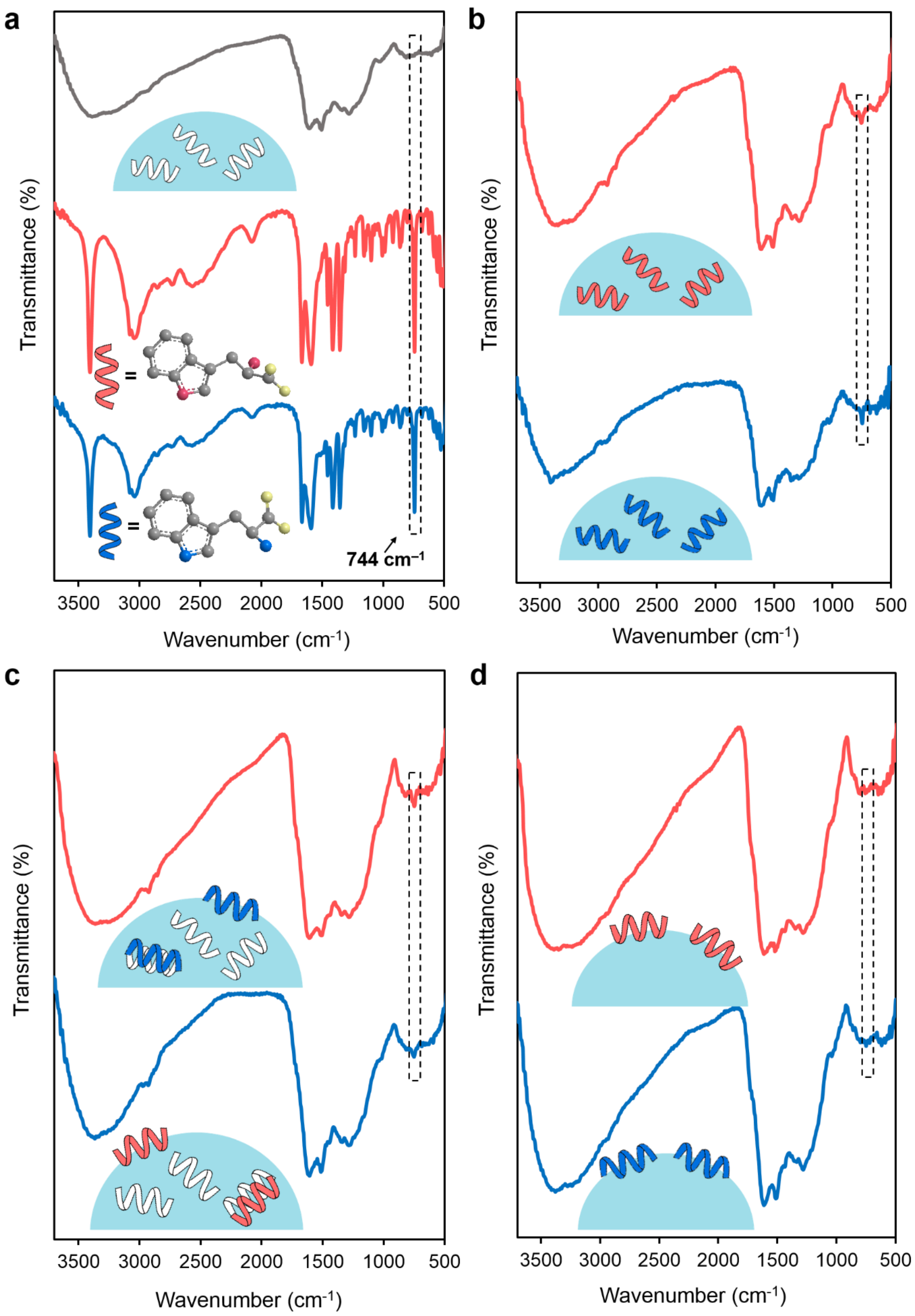

Supplementary Figure 9. **Study of binding type by FT-IR.** FT-IR spectra of **a** D-Tryptophan (blue), L-Tryptophan (pink), and CIPPs (grey) **b** D-IPPs (blue) and L-IPPs (pink) after recognition of D-Tryptophan and L-Tryptophan, respectively, **c** D-IPPs (blue) and L-IPPs (pink) after recognition of L-Tryptophan and D-Tryptophan, respectively, and **d** NIPPs after recognition of D-Tryptophan (blue) and L-Tryptophan (pink). The inset in **a** is the molecular structure of Tryptophan enantiomers and schematic of CIPPs. The insets in **b-d** are CIPPs or NIPPs and bonded enantiomers' status.

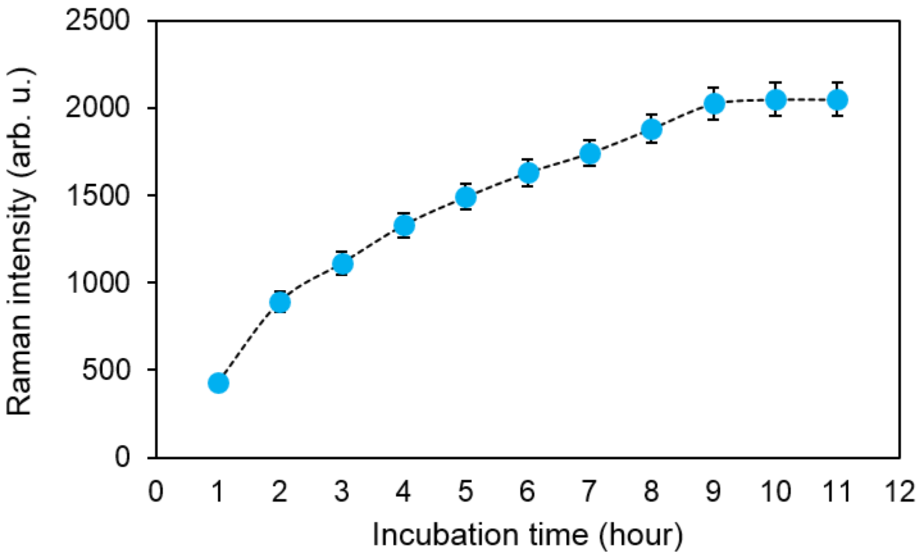

Supplementary Figure 10. **Platform construction optimization.** Effect of Au NSs incubation time on the Raman spectra of DTTC. Error bars represent the standard deviations (n = 3). Source data are provided as a Source Data file.

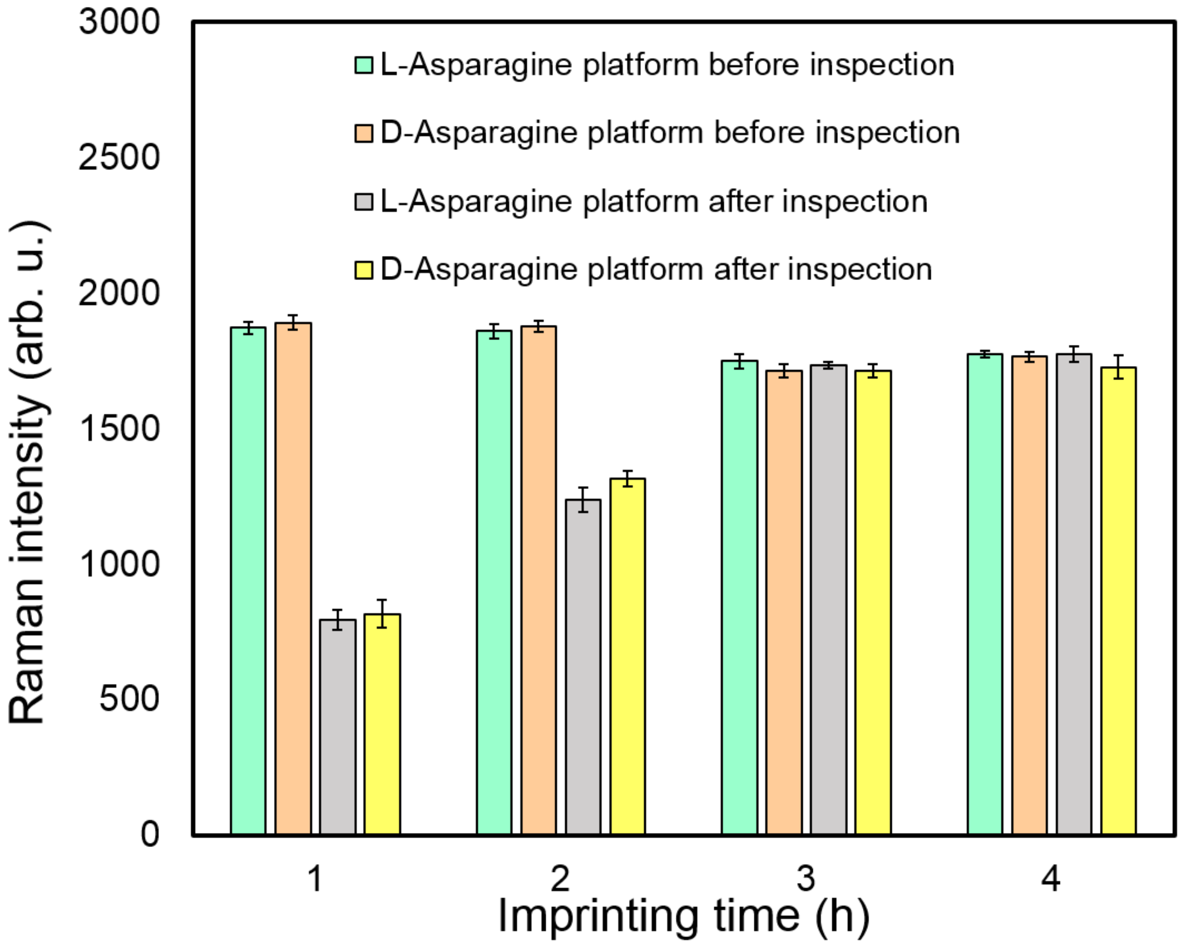

Supplementary Figure 11. **Platform construction optimization.** The diagram effect of surface imprinting time versus SERS intensity of DTTC before and after inspector inspection. Error bars represent the standard deviations (n = 3). Source data are provided as a Source Data file.

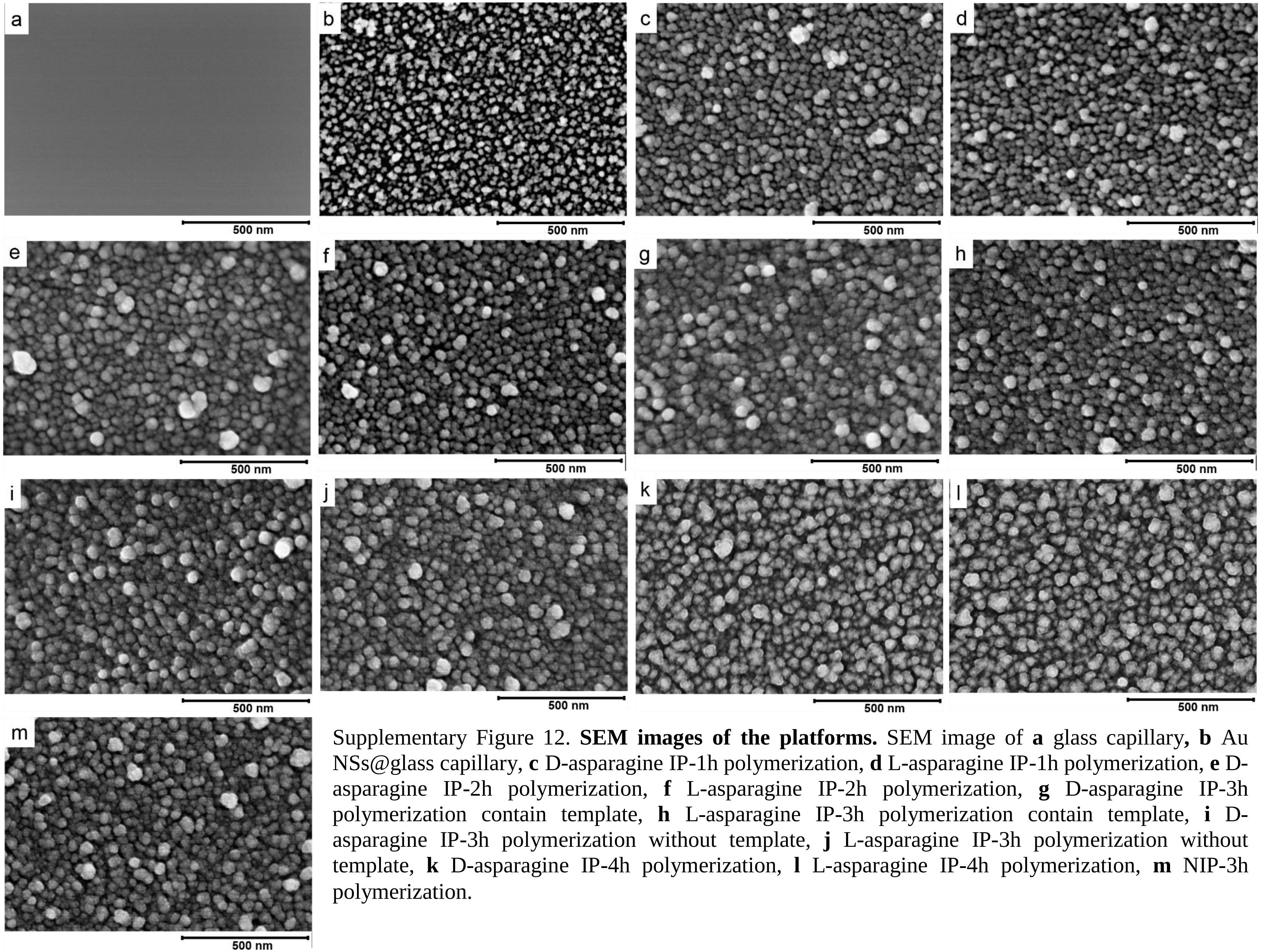

Supplementary Figure 12. **SEM images of the platforms.** SEM image of **a** glass capillary**, b** Au NSs@glass capillary, **c** D-asparagine IP-1h polymerization, **d** L-asparagine IP-1h polymerization, **e** D-asparagine IP-2h polymerization, **f** L-asparagine IP-2h polymerization, **g** D-asparagine IP-3h polymerization contain template, **h** L-asparagine IP-3h polymerization contain template, **i** D-asparagine IP-3h polymerization without template, **j** L-asparagine IP-3h polymerization without template, **k** D-asparagine IP-4h polymerization, **l** L-asparagine IP-4h polymerization, **m** NIP-3h polymerization.

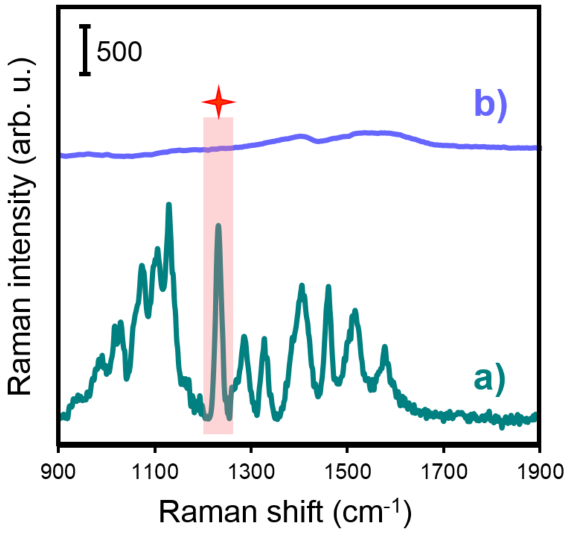

Supplementary Figure 13. **Degradation of DTTC by cysteamine on the SERS tag@capillary.** Raman spectra of DTTC on the SERS tag@capillary **a** before, and **b** after incubation in cysteamine solution (0.5 M, incubation time of one second). Source data are provided as a Source Data file.

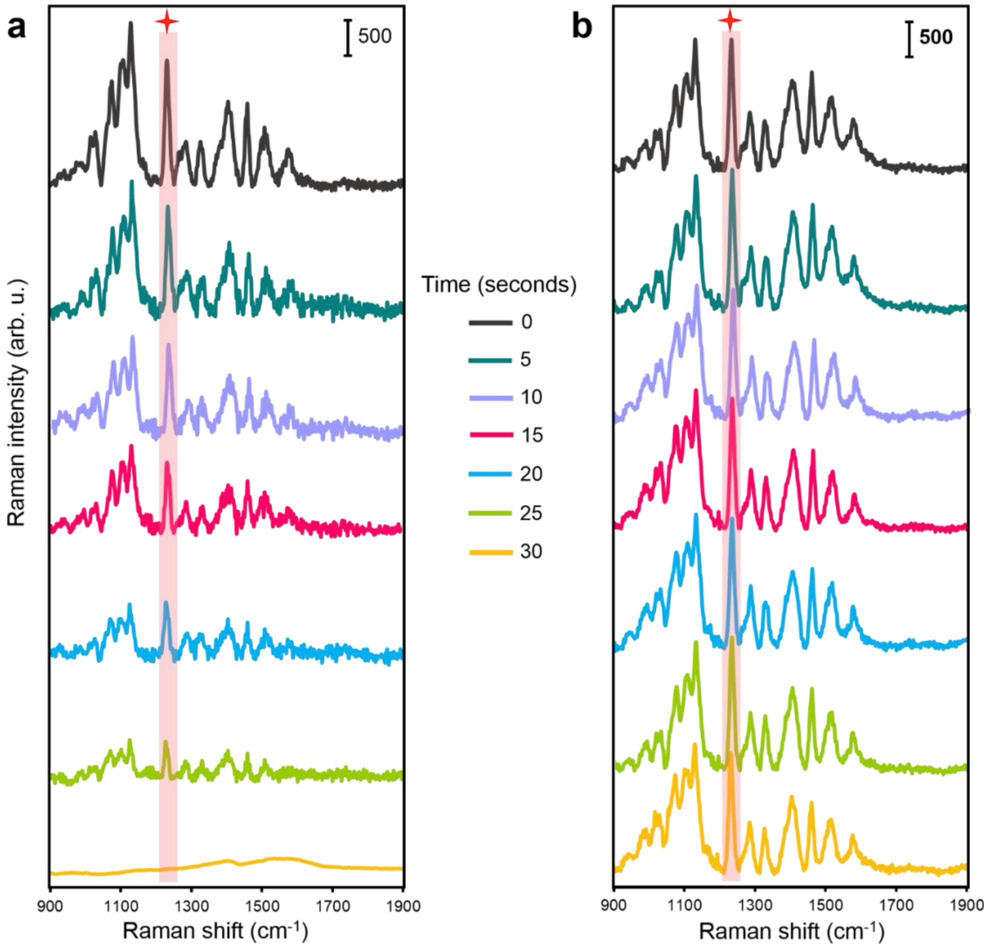

Supplementary Figure 14. **Kinetic of cysteamine diffusion.** Raman spectra of DTTC on the **a** SERS-CIP, and **b** SERS-NIP incubated in cysteamine solution (0.5 M) at different time. Source data are provided as a Source Data file.

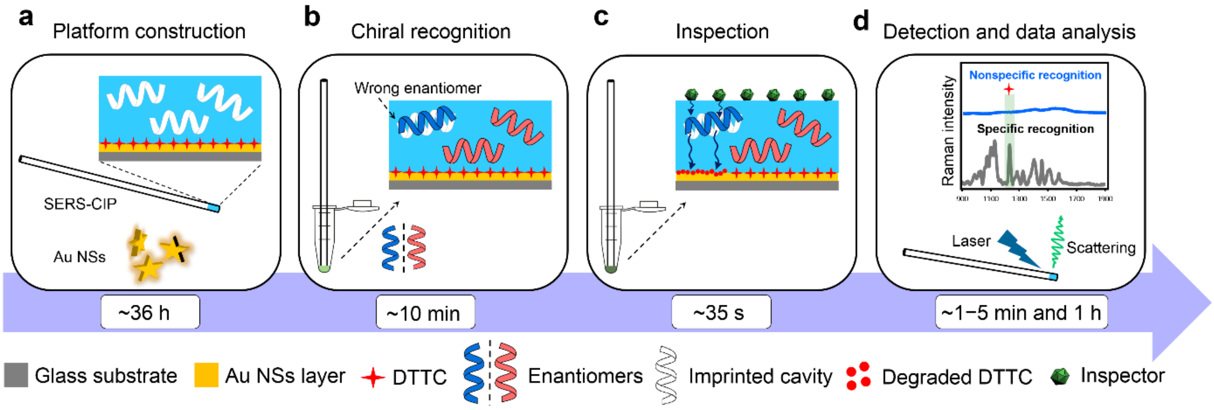

Supplementary Figure 15. **Overview of the IRM procedure.** Overview of the IRM procedure for absolute chiral discrimination. **a** Fabrication of the materials used for IRM, including SERS tag@capillary and forming chiral imprinted PDA on the surface of SERS tag@capillary. **b** Chiral recognition by a SERS-CIP just dipped into a solution under test. **c** Inspection of imprinted cavities by diffusion of inspector molecules through the vacant and nonspecifically occupied cavities and degradation of DTTC molecules under the aforementioned cavities. **d** Raman signal readout by plasmonic detection and data analysis.

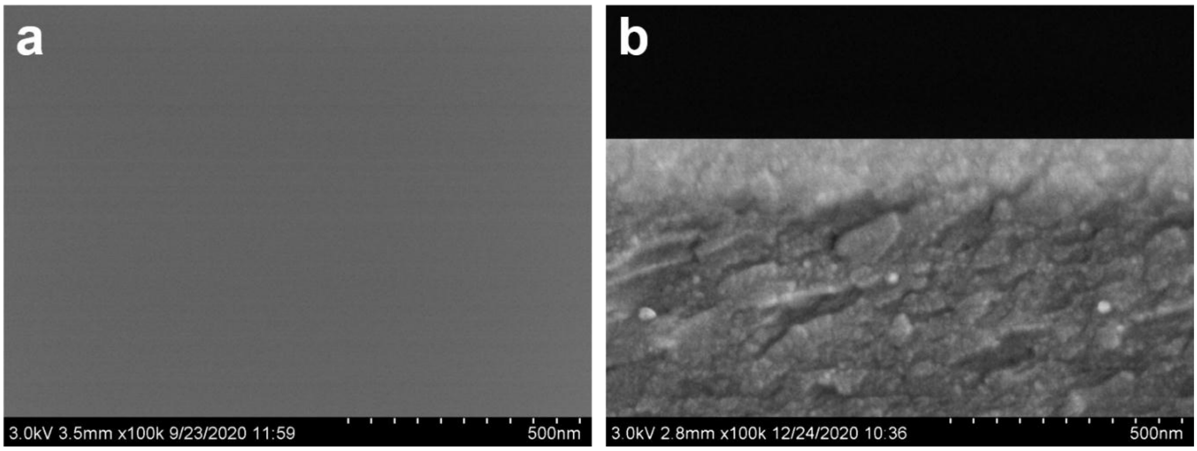

Supplementary Figure 16. **The smoothness of a glass capillary surface. a** SEM and **b** cross-SEM images of glass capillary.

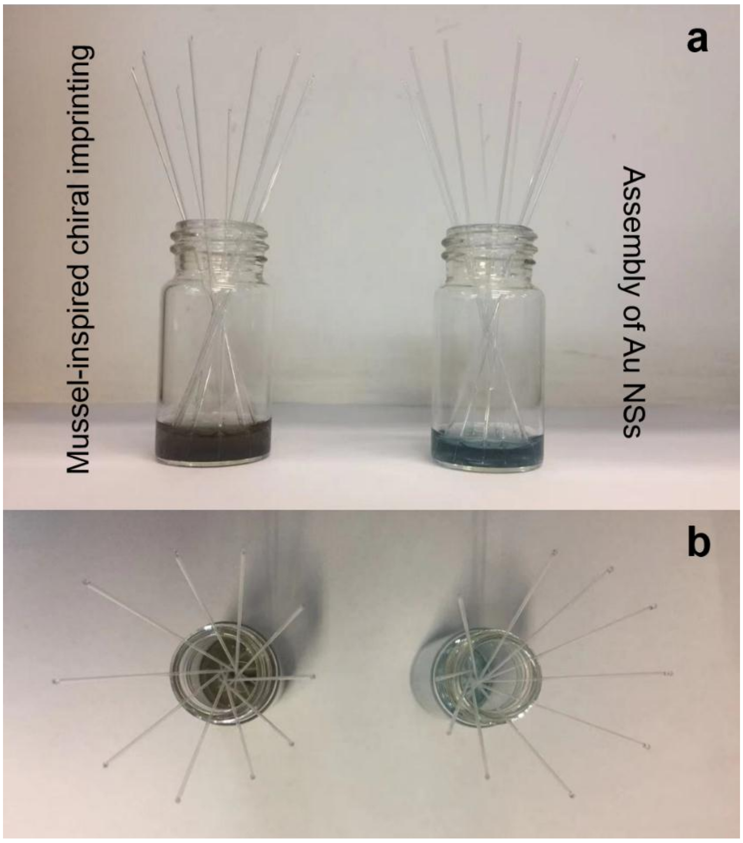

Supplementary Figure 17. **Scalability of the platforms' construction.** Photo images of a batch of SERS-CIPs construction. **a** Front view of Au Ns immobilization on the surface of amino-functionalized glass capillaries and mussel-inspired chiral imprinting. **b** Top view.

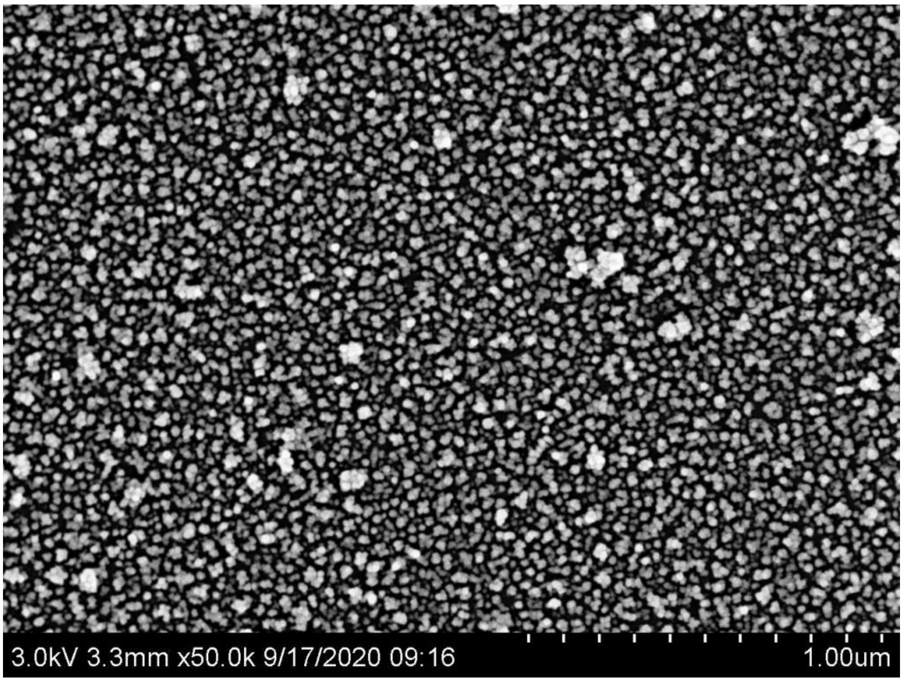

Supplementary Figure 18. **Uniformity of Au NSs immobilization.** SEM image of Au NSs assembled on capillary glass.

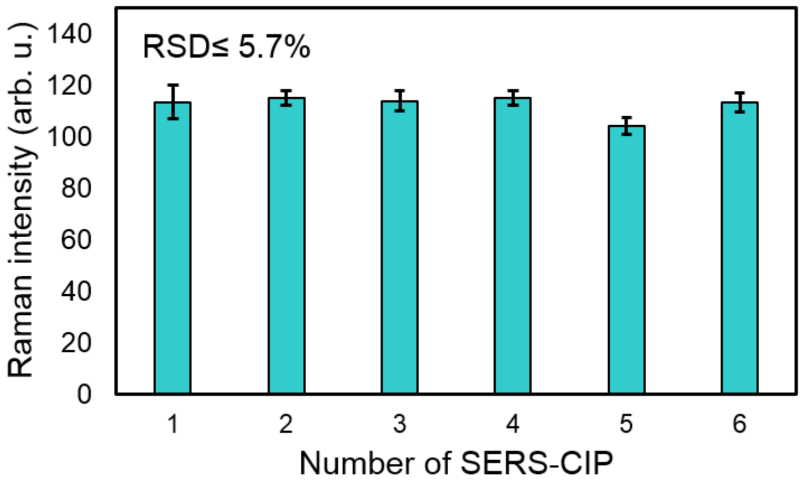

Supplementary Figure 19. **Reproducibility of SERS signals.** Distributions of Raman peak intensity at 1246 cm^{-1} for six SERS-CIP. The RSDs for six SERS-CIP were not higher than 5.7%. Raman laser power was 5 mW. Source data are provided as a Source Data file.

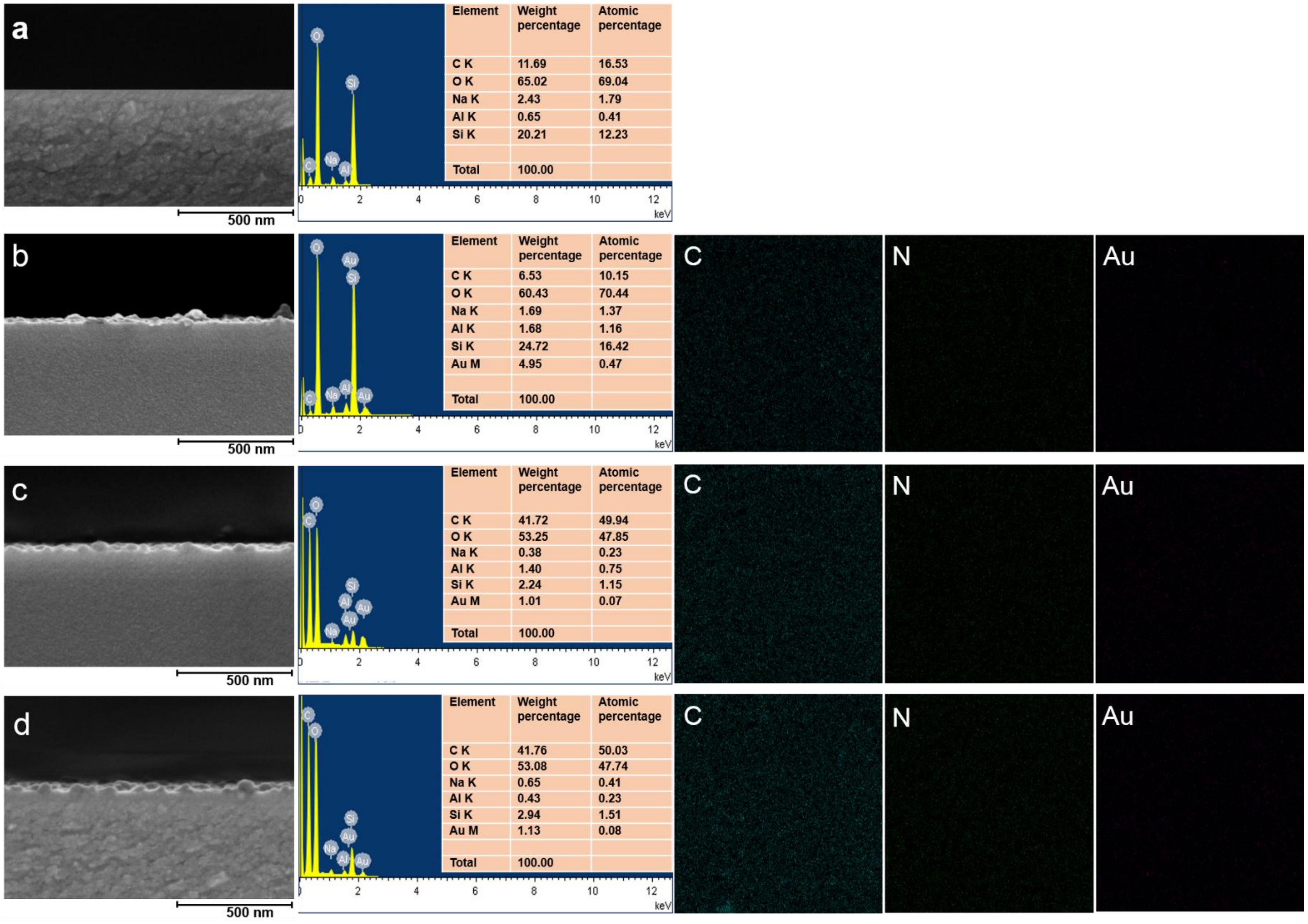

Supplementary Figure 20. **Morphology and elemental composition of the platforms.** The cross-sectional SEM, EDX analysis, and EDX mapping of **a** bare glass capillary, **b** glass capillary@Au NSs, **c** SERS-L-IP, and **d** SERS-D-IP.

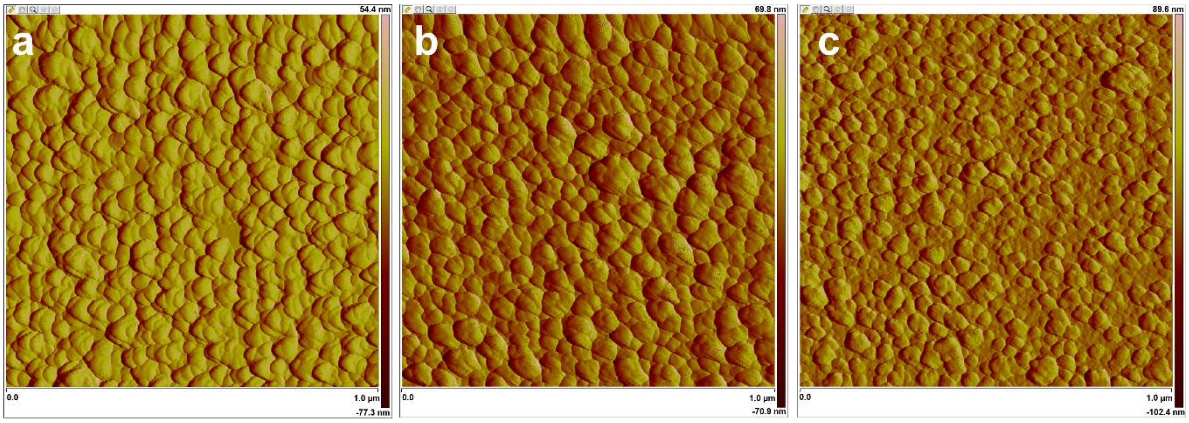

Supplementary Figure 21. **Morphology of the platforms.** AFM image of the **a** glass capillary@Au NSs, **b** SERS-L-IP, and **c** SERS-D-IP.

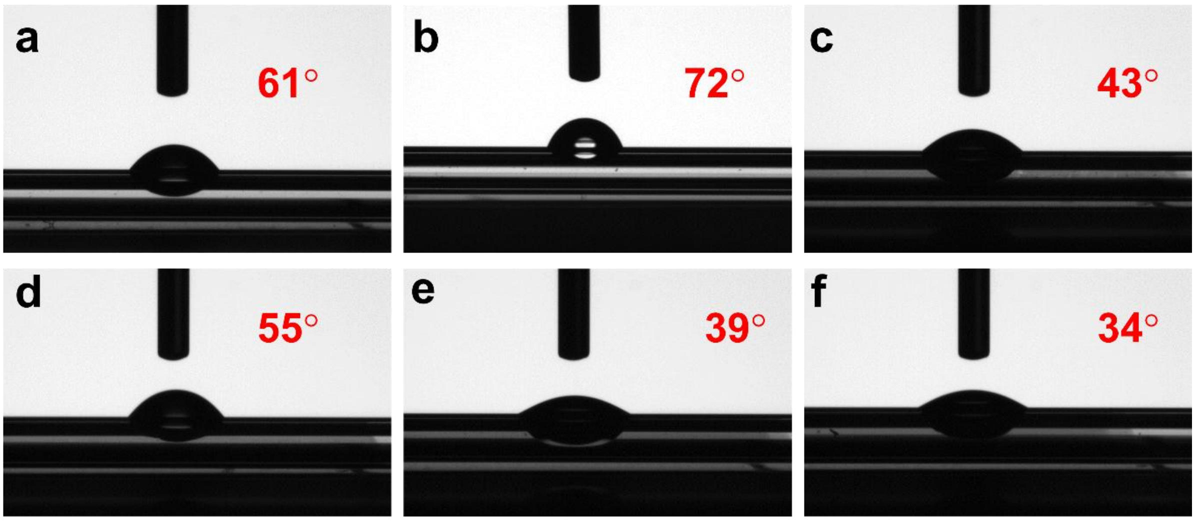

Supplementary Figure 22. **Hydrophilicity of the platforms.** Water contact angle of the **a** bare glass capillary, **b** amino-functionalized glass capillary, **c** glass capillary@Au NSs, **d** SERS tag@capillary, **e** SERS-L-IP, and **f** SERS-D-IP.

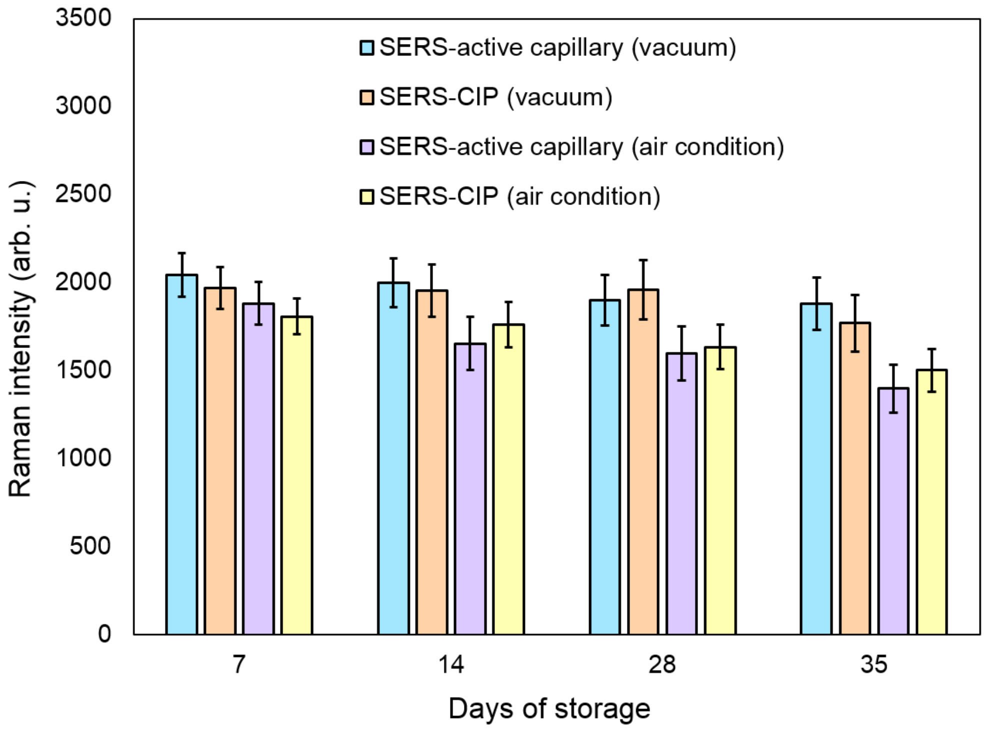

Supplementary Figure 23. **Stability of the platforms.** SERS intensities of SERS-active glass capillary and SERS-CIP within 35 days of storage in a vacuum and ambient conditions. Error bars represent the standard deviations (n = 3). Source data are provided as a Source Data file.

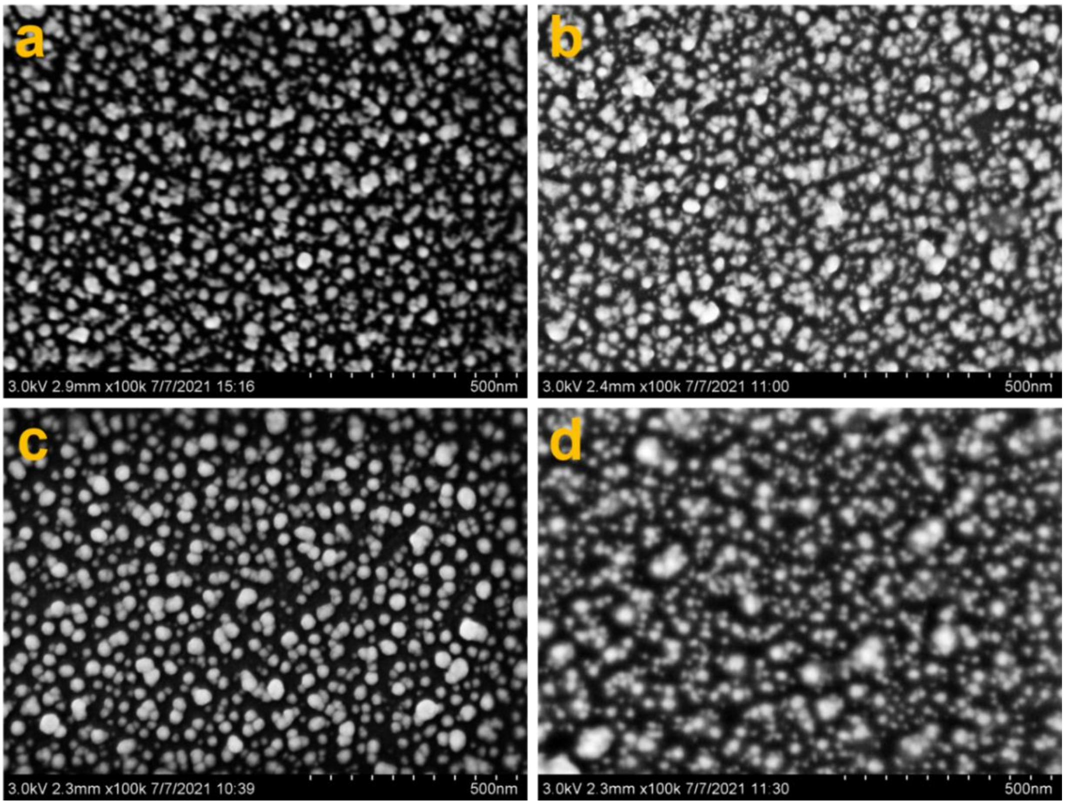

Supplementary Figure 24. **Stability of the platforms.** The SEM images of **a** and **b** SERS-active glass capillary, **c** and **d** SERS-CIP after 35 days of storage in vacuum and ambient conditions, respectively.

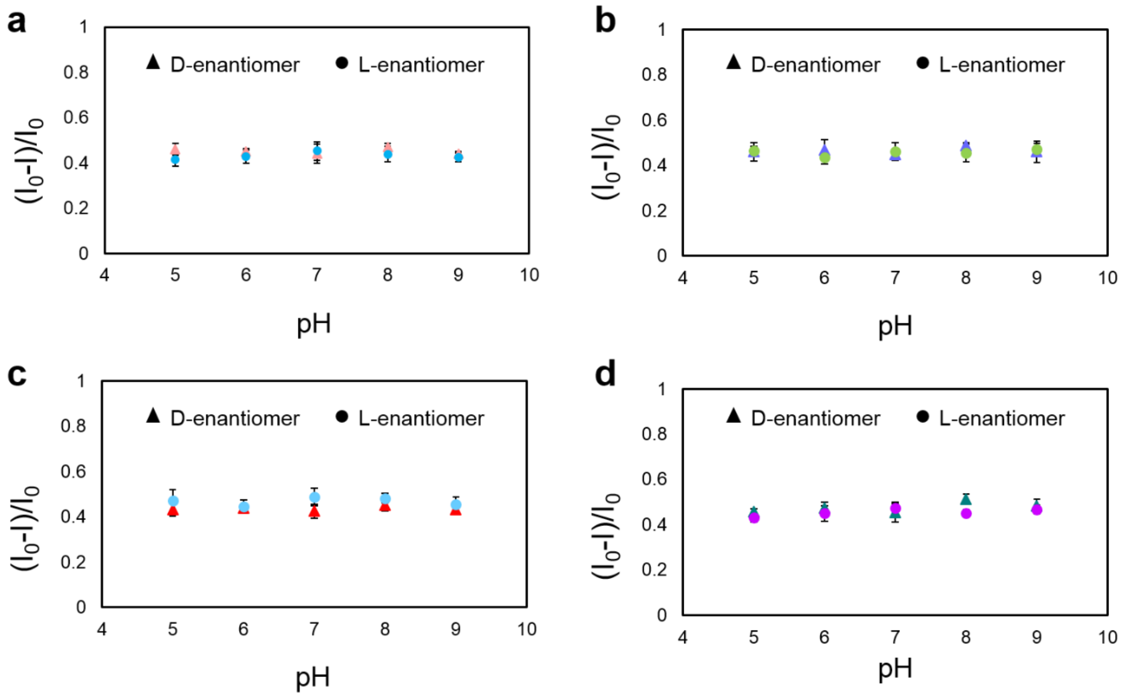

Supplementary Figure 25. **Chiral recognition dependency to pH.** Effect of pH on the recognition performance of **a** arginine, **b** asparagine, **c** histidine, **d** tryptophan SERS-CIPs. pH was adjusted by 0.01 M phosphate buffer. Error bars represent the standard deviations (n = 3). Source data are provided as a Source Data file.

Supplementary Tables

Supplementary Table 1. **Method comparison.** Different approaches for chiral recognition of Tryptophan and Histidine as chiral models.

Receptor	Chiral target	Mechanism	Detection scheme	Response range	Enantio sensitivity	Ref.
Dye anthracene labeled amylose-functionalized graphene	Tryptophan	Different fluorescence recoveries	Fluorescence	0–5 μM	LODs are 134 and 34 nM for D and L-Tryptophan, respectively.	48
β-cyclodextrin capped Mn-doped ZnS QDs	Tryptophan	Selective hydrolysis followed by fluorescence enhancement	Fluorescence	0–6.0 μM	LOD for L-Tryptophan is 5.4 nM	49
L/D-Cysteine modified CDs	Tryptophan	Different fluorescence quenching	Fluorescence	0–2.8 mM	-	50
Black phosphorus@graphene nanomaterial	Tryptophan	Enhancement of Raman signal based on a chemical mechanism	SERS	0.48–4.8 μM	-	51
Mono (6-mercapto-6-deoxy)-beta-cyclodextrin combined with p-Aminothiophenol	Tryptophan	Charge transfer	SERS	10^{-8}–10^{-3} M	The minimum enantiomeric discrimination concentration for L-Tryptophan is 0.1 μM	52
The synergy of chiral ionic liquid and three-dimensional N-doped graphene oxide multi-walled carbon nanotubes	Tryptophan	Noncovalent interactions followed by the difference of the peak current response	Electrochemical	0.01–5 mM	LODs are 0.055 and 0.024 μM for D and L-Tryptophan, respectively.	53
Nonionic surfactant-assisted	Tryptophan	Recognition by chiral imprinted	Electrochemical	0.2–4.0 mM	LODs are 1.27×10^{-4} and 1.06×10^{-4}	54

molecular imprinting silica		cavities			M for D and L-Tryptophan, respectively.	
Homochiral metal–organic framework	Tryptophan	Static quenching and competitive absorption	Fluorescence	-	LODs are 2.59 and 1.94 μM for D and L-Tryptophan, respectively.	55
CIP	Tryptophan	IRM	SERS	4.9×10^{-12}–4.8×10^{-6} M	LODs are 2.4×10^{-13} and 5.4×10^{-13} M for L and D- Tryptophan, respectively.	This work
Titanium dioxide nanochannel arrays/β-cyclodextrin	Histidine	Adsorbing hydrophobic imidazole ring of L-His into the β-cyclodextrin cavity and incident light as an exciting resource and electrical current as a readout signal	Photoelectrochemical	0.2–1.0 mM	67.5 μM	56
Poly(2-oxazoline) derivatives bearing chiral pyrrolidine–triazole moieties in the side chain	Histidine	nitrogen/Cu2+ coordination forms complexes exhibiting induced circular dichroism signals	Circular dichroism	0.5×10^{-5}–5.0×10^{-5} M	10 μM	57
Molecularly imprinted polymer	Histidine	Recognition by chiral imprinted cavities	Electrochemical	100 nM–10 μM	LODs are 10 and 100 nM for D and L- Histidine, respectively	58
CIP	Histidine	IRM	SERS	6.4×10^{-12}–6.4×10^{-6} M	LODs are 7×10^{-13} and 1.7×10^{-13} M for L and D- Histidine, respectively.	This work

Supplementary Table 2. **Practical application of SERS-CIP.** Application of the SERS-CIP for the enantiodetermination of chiral compounds in seawater and urine samples (n = 4).

	Add (μg L^{-1})	Found (μg L^{-1}) ± SD	Recovery (%)	Add (μg L^{-1})	Found (μg L^{-1}) ± SD	Recovery (%)
	Seawater			Urine		
L-asparagine	0	N.D	-	0	N.D	-
	10	8.4 ± 0.9	84.8	10	10.3 ± 2.0	103.3
	100	102.1 ± 25.0	102.1	100	88.6 ± 16.1	88.6
D-asparagine	0	N.D	-	0	N.D	-
	10	9.2 ± 1.7	92.9	10	9.6 ± 2.4	96.5
	100	82.8 ± 15.8	82.8	100	90.6 ± 17.0	90.6
L-galactose	0	N.D	-	0	N.D	-
	10	9.7 ± 2.3	97.1	10	8.9 ± 0.9	89.9
	100	101.8 ± 22.2	101.8	100	91.3 ± 26.2	91.3
D-galactose	0	N.D	-	0	N.D	-
	10	9.4 ± 1.9	94.6	10	10.8 ± 2.7	108.4
	100	95.5 ± 12.4	95.5	100	88.3 ± 16.2	88.3

N.D = Not detected.

Supplementary Table 3. **Surface charge of PDA.** Zeta potential (mv) of PDA and amino acids at different pH. pH was adjusted by 0.01 M phosphate buffer.

Compound	pH=5.0	pH=6.0	pH=7.0	pH=8.0	pH=9.0
PDA	+4.9	-8.0	-14.9	-32.5	-24.8
L-Arginine	-15.3	-13.3	-13.6	-7.4	-14.3
D-Arginine	-16.7	-14.3	-7.5	-7.8	-7.8
L-Asparagine	-11.8	-25.2	-11.6	-6.91	-7.51
D-Asparagine	-7.6	-19.9	-16.9	-27.4	-30.0
L-Histidine	-13.2	-17.9	-21.8	-15.3	-17.6
D-Histidine	-18.5	-20.2	-18.1	-13.5	-12.7
L-Tryptophan	-10.6	-7.0	9.7	8.5	11.2
D-Tryptophan	-4.2	-8.0	-6.8	-11.8	-15.7

Supplementary References

1. Zangiabadi M, Zhao Y. Selective binding of complex glycans and glycoproteins in water by molecularly imprinted nanoparticles. *Nano Lett.* **20**, 5106-5110 (2020).
2. Zhang Z, Zhang X, Liu B, Liu J. Molecular imprinting on inorganic nanozymes for hundred-fold enzyme specificity. *J. Am. Chem. Soc.* **139**, 5412-5419 (2017).
3. Liu W, et al. Complete mapping of DNA-protein interactions at the single-molecule level. *Adv. Sci.* e2101383 (2021).
4. Gunasekara RW, Zhao Y. A general method for selective recognition of monosaccharides and oligosaccharides in water. *J. Am. Chem. Soc.* **139**, 829-835 (2017).
5. Awino JK, Gunasekara RW, Zhao Y. Sequence-selective binding of oligopeptides in water through hydrophobic coding. *J. Am. Chem. Soc.* **139**, 2188-2191 (2017).
6. Sellergren B, Lepistoe M, Mosbach K. Highly enantioselective and substrate-selective polymers obtained by molecular imprinting utilizing noncovalent interactions. NMR and chromatographic studies on the nature of recognition. *J. Am. Chem. Soc.* **110**, 5853-5860 (1988).
7. Fischer L, Mueller R, Ekberg B, Mosbach K. Direct enantioseparation of beta-adrenergic blockers using a chiral stationary phase prepared by molecular imprinting. *J. Am. Chem. Soc.* **113**, 9358-9360 (1991).
8. Fireman-Shoresh S, Avnir D, Marx S. General method for chiral imprinting of sol−gel thin films exhibiting enantioselectivity. *Chem. Mater.* **15**, 3607-3613 (2003).
9. Cao X, Fischer G. Infrared spectral, structural, and conformational studies of zwitterionic L-tryptophan. *J. Phys. Chem. A* **103**, 9995-10003 (1999).
10. Leyton P, Brunet J, Silva V, Paipa C, Castillo MV, Brandán SA. An experimental and theoretical study of l-tryptophan in an aqueous solution, combining two-layered ONIOM and SCRF calculations. *Spectrochim. Acta A Mol. Biomol. Spectrosc.* **88**, 162-170 (2012).
11. Ryu JH, Messersmith PB, Lee H. Polydopamine surface chemistry: a decade of discovery. *ACS Appl. Mater. Interfaces* **10**, 7523-7540 (2018).
12. Lee HA, Park E, Lee H. Polydopamine and its derivative surface chemistry in material science: a focused review for studies at KAIST. *Adv. Mater.* **32**, 1907505 (2020).
13. Liu Y, Ai K, Lu L. Polydopamine and its derivative materials: synthesis and promising applications in energy, environmental, and biomedical fields. *Chem. Rev.* **114**, 5057-5115 (2014).
14. Della Vecchia NF, et al. Tris buffer modulates polydopamine growth, aggregation, and paramagnetic properties. *Langmuir* **30**, 9811-9818 (2014).
15. Ju KY, Lee Y, Lee S, Park SB, Lee JK. Bioinspired polymerization of dopamine to generate melanin-like nanoparticles having an excellent free-radical-scavenging property. *Biomacromolecules* **12**, 625-632 (2011).
16. Bernsmann F, et al. Dopamine−melanin film deposition depends on the used oxidant and buffer solution. *Langmuir* **27**, 2819-2825 (2011).
17. Liu X, et al. Mussel-inspired polydopamine: a biocompatible and ultrastable coating for nanoparticles in vivo. *ACS Nano* **7**, 9384-9395 (2013).
18. Lee H, Dellatore SM, Miller WM, Messersmith PB. Mussel-inspired surface chemistry for multifunctional coatings. *Science* **318**, 426-430 (2007).

19. Liu J, He H, Xie D, Wen Y, Liu Z. Probing low-copy-number proteins in single living cells using single-cell plasmonic immunosandwich assays. *Nat. Protoc.* **16**, 3522-3546 (2021).
20. Xing R, Ma Y, Wang Y, Wen Y, Liu Z. Specific recognition of proteins and peptides via controllable oriented surface imprinting of boronate affinity-anchored epitopes. *Chem. Sci.* **10**, 1831-1835 (2019).
21. Chen L, Wang X, Lu W, Wu X, Li J. Molecular imprinting: perspectives and applications. *Chem. Soc. Rev.* **45**, 2137-2211 (2016).
22. Lofgreen JE, Ozin GA. Controlling morphology and porosity to improve performance of molecularly imprinted sol–gel silica. *Chem. Soc. Rev.* **43**, 911-933 (2014).
23. Arabi M, et al. Molecular Imprinting: Green perspectives and strategies. *Adv. Mater.* **33**, 2100543 (2021).
24. Cheng W, et al. Versatile polydopamine platforms: synthesis and promising applications for surface modification and advanced nanomedicine. *ACS Nano* **13**, 8537-8565 (2019).
25. Luo J, Jiang S, Liu X. Efficient one-pot synthesis of mussel-inspired molecularly imprinted polymer coated graphene for protein-specific recognition and fast separation. *J. Phys. Chem. C* **117**, 18448-18456 (2013).
26. Li JF, et al. Shell-isolated nanoparticle-enhanced Raman spectroscopy. *Nature* **464**, 392-395 (2010).
27. Wang Y, Zhao X, Yu Z, Xu Z, Zhao B, Ozaki Y. A chiral-label-free SERS strategy for the synchronous chiral discrimination and identification of small aromatic molecules. *Angew. Chem. Int. Ed.* **132**, 19241-19248 (2020).
28. Zhang W, et al. Plasmonic chiral metamaterials with sub-10 nm nanogaps. *ACS Nano* **15**, 17657-17667 (2021).
29. Jimenez de Aberasturi D, Serrano-Montes AB, Langer J, Henriksen-Lacey M, Parak WJ, Liz-Marzan LM. Surface enhanced Raman scattering encoded gold nanostars for multiplexed cell discrimination. *Chem. Mater.* **28**, 6779-6790 (2016).
30. Rodr ǵuez-Lorenzo L, et al. Zeptomol detection through controlled ultrasensitive surface-enhanced Raman scattering. *J. Am. Chem. Soc.* **131**, 4616-4618 (2009).
31. Wang Y, Yan B, Chen L. SERS tags: novel optical nanoprobes for bioanalysis. *Chem. Rev.* **113**, 1391-1428 (2013).
32. Li M, Kang JW, Dasari RR, Barman I. Shedding light on the extinction-enhancement duality in gold nanostar-enhanced Raman spectroscopy. *Angew. Chem. Int. Ed.* **126**, 14339-14343 (2014).
33. Lee M, et al. Subnanomolar sensitivity of filter paper-based SERS sensor for pesticide detection by hydrophobicity change of paper surface. *ACS Sens.* **3**, 151-159 (2018).
34. Zhang K, Liu Y, Wang Y, Zhao J, Liu B. Direct SERS tracking of a chemical reaction at a single 13 nm gold nanoparticle. *Chem. Sci.* **10**, 1741-1745 (2019).
35. Wang H, et al. Simultaneous capture, detection, and inactivation of bacteria as enabled by a surface-enhanced Raman scattering multifunctional chip. *Angew. Chem. Int. Ed.* **127**, 5221-5225 (2015).
36. Zhou B, et al. Amphiphilic functionalized acupuncture needle as SERS sensor for in situ multiphase detection. *Anal. Chem.* **90**, 3826-3832 (2018).

37. Arabi M, et al. Label-free SERS detection of Raman-inactive protein biomarkers by Raman reporter indicator: Toward ultrasensitivity and universality. *Biosens. Bioelectron.* **174**, 112825 (2021).
38. Su Q, Ma X, Dong J, Jiang C, Qian W. A reproducible SERS substrate based on electrostatically assisted APTES-functionalized surface-assembly of gold nanostars. *ACS Appl. Mater. Interfaces* **3**, 1873-1879 (2011).
39. Grabar KC, et al. Kinetic control of interparticle spacing in Au colloid-based surfaces: rational nanometer-scale architecture. *J. Am. Chem. Soc.* **118**, 1148-1153 (1996).
40. Wang Y, Qian W, Tan Y, Ding S. A label-free biosensor based on gold nanoshell monolayers for monitoring biomolecular interactions in diluted whole blood. *Biosens. Bioelectron.* **23**, 1166-1170 (2008).
41. Zhang M, et al. Gold-trisoctahedra-coated capillary-based SERS platform for microsampling and sensitive detection of trace fentanyl. *Anal. Chem.* **94**, 4850-4858 (2022).
42. Nguyen TD, Song MS, Ly NH, Lee SY, Joo SW. Nanostars on nanopipette tips: A Raman probe for quantifying oxygen levels in hypoxic single cells and tumours. *Angew. Chem. Int. Ed.* **131**, 2736-2740 (2019).
43. Xi W, Haes AJ. Elucidation of HEPES affinity to and structure on gold nanostars. *J. Am. Chem. Soc.* **141**, 4034-4042 (2019).
44. Webb JA, et al. Geometry-dependent plasmonic tunability and photothermal characteristics of multibranched gold nanoantennas. *J. Phys. Chem. C* **118**, 3696-3707 (2014).
45. Xianyu Y, Lin Y, Chen Q, Belessiotis-Richards A, Stevens MM, Thomas MR. Iodide-mediated rapid and sensitive surface etching of gold nanostars for biosensing. *Angew. Chem. Int. Ed.* **133**, 9979-9984 (2021).
46. Zhang X, Sucre-Rosales E, Byram A, Hernandez FE, Chen G. Ultrasensitive visual detection of glucose in urine based on the iodide-promoted etching of gold bipyramids. *ACS Appl. Mater. Interfaces* **12**, 49502-49509 (2020).
47. Wu D, et al. Surface molecular imprinting over supported metal catalysts for size-dependent selective hydrogenation reactions. *Nat. Catal.* **4**, 595-606 (2021).
48. Wei W, Qu K, Ren J, Qu X. Chiral detection using reusable fluorescent amylose-functionalized graphene. *Chem. Sci.* **2**, 2050-2056 (2011).
49. Wei Y, Li H, Hao H, Chen Y, Dong C, Wang G. β-Cyclodextrin functionalized Mn-doped ZnS quantum dots for the chiral sensing of tryptophan enantiomers. *Polym. Chem.* **6**, 591-598 (2015).
50. Askari F, Rahdar A, Trant JF. L-tryptophan adsorption differentially changes the optical behaviour of pseudo-enantiomeric cysteine-functionalized quantum dots: Towards chiral fluorescent biosensors. *Sens. Biosensing Res.* **22**, 100251 (2019).
51. Ranc V, Chaloupková Z. Chiral discrimination of amino acids using phosphorene assisted graphene-enhanced Raman spectroscopy. *Anal. Chim. Acta* **1129**, 69-75 (2020).
52. Wang Y, et al. A chiral signal-amplified sensor for enantioselective discrimination of amino acids based on charge transfer-induced SERS. *Chem. Commun.* **55**, 9697-9700 (2019).

53. Liu N, Liu J, Niu X, Wang J, Guo R, Mo Z. An electrochemical chiral sensor based on the synergy of chiral ionic liquid and 3D-NGMWCNT for tryptophan enantioselective recognition. *Microchim. Acta* **188**, 1-13 (2021).
54. Li Z, et al. Electrochemical chiral recognition of tryptophan isomers based on nonionic surfactant-assisted molecular imprinting sol–gel silica. *ACS Appl. Mater. Interfaces* **11**, 2840-2848 (2018).
55. Zhu Y, Zhou Y, Zhang X, Sun Z, Jiao C. Homochiral MOF as chiroptical sensor for determination of absolute configuration and enantiomeric ratio of chiral tryptophan. *Adv. Opt. Mater.* **9**, 2001889 (2021).
56. Liu J, Fu B, Zhang Z. Ionic current rectification triggered photoelectrochemical chiral sensing platform for recognition of amino acid enantiomers on self-standing nanochannel arrays. *Anal. Chem.* **92**, 8670-8674 (2020).
57. Liu K, Du G, Ye L, Jiang L. A chiroptical nanoprobe for highly selective recognition of histidine enantiomers in aqueous media. *Sens. Actuators B Chem.* **284**, 55-62 (2019).
58. Zhang L, et al. Selective recognition of Histidine enantiomers using novel molecularly imprinted organic transistor sensor. *Org. Electron.* **61**, 254-260 (2018).
