# Supplementary material for: Chiral molecular imprinting-based SERS detection strategy for absolute enantiomeric discrimination
Source: Nat Commun. 2022 Sep 30;13:5757. doi: 10.1038/s41467-022-33448-w (PMC9525700; doi:10.1038/s41467-022-33448-w)
Supplement: Supplementary file 2 — Reporting Summary [file 41467_2022_33448_MOESM2_ESM.pdf]

nature portfolio

Corresponding author(s): Yunqing Wang, Lingxin Chen

Last updated by author(s): Sep 9, 2022

Reporting Summary

Nature Portfolio wishes to improve the reproducibility of the work that we publish. This form provides structure for consistency and transparency in reporting. For further information on Nature Portfolio policies, see our Editorial Policies and the Editorial Policy Checklist.

Statistics

For all statistical analyses, confirm that the following items are present in the figure legend, table legend, main text, or Methods section.

n/a	Confirmed	
☐	☒	The exact sample size (*n*) for each experimental group/condition, given as a discrete number and unit of measurement
☐	☒	A statement on whether measurements were taken from distinct samples or whether the same sample was measured repeatedly
☐	☒	The statistical test(s) used AND whether they are one- or two-sided<br>*Only common tests should be described solely by name; describe more complex techniques in the Methods section.*
☐	☒	A description of all covariates tested
☐	☒	A description of any assumptions or corrections, such as tests of normality and adjustment for multiple comparisons
☐	☒	A full description of the statistical parameters including central tendency (e.g. means) or other basic estimates (e.g. regression coefficient) AND variation (e.g. standard deviation) or associated estimates of uncertainty (e.g. confidence intervals)
☒	☐	For null hypothesis testing, the test statistic (e.g. *F*, *t*, *r*) with confidence intervals, effect sizes, degrees of freedom and *P* value noted<br>*Give P values as exact values whenever suitable.*
☒	☐	For Bayesian analysis, information on the choice of priors and Markov chain Monte Carlo settings
☐	☒	For hierarchical and complex designs, identification of the appropriate level for tests and full reporting of outcomes
☒	☐	Estimates of effect sizes (e.g. Cohen's *d*, Pearson's *r*), indicating how they were calculated

Our web collection on statistics for biologists contains articles on many of the points above.

Software and code

Policy information about availability of computer code

Data collection	Scanning electron microscopy (SEM) images were recorded by an S-4800 field emission scanning electron microscope (Hitachi, Japan). Transmission electron microscopy (TEM) images were obtained on a JEM-1400 transmission electron microscope (JEOL, Japan). Atomic force microscopy (AFM) images were obtained on an atomic force microscope (Veeco, USA). Static water contact angles were measured using an OCA50 system (Dataphysics, Germany). Circular dichroism (CD) spectra were collected on the Chirascan spectropolarimeter. Isothermal titration calorimetry (ITC) was performed using a Nano calorimeter (Waters, TA Instruments, USA). FT-IR spectra obtained from a Fourier transform infrared spectrometer (Nicolet IS10, Thermo Fisher). Zeta potential was detected on Malvern Zetasizer Nano-ZS90 (ZEN3590, UK). The UV–Vis absorption spectra were recorded on a Thermo Scientific NanoDrop 2000/2000C spectrophotometer. Fluorescence spectra were recorded on a HORIBA Scientific Fluoromax-4 spectrofluorometer. The SERS spectra were collected on a DXR Raman microscope (Thermo Scientific, USA). The excitation light source was a He–Ne laser operating at λ = 780 nm, and the laser spot was focused on the platform through a 10× objective lens. The baseline correction of Raman spectra was conducted using OMNIC for dispersive Raman 8.3.104 series software (Thermo Fisher Scientific Inc.).
Data analysis	SERS: OMNIC for dispersive Raman 8.3.104 series software (Thermo Fisher Scientific Inc.)<br>SEM: PC-SEM 3.18.0.3 (HITACHI)<br>TEM: TEM Center 0210 (JEOL)<br>AFM: Nanoscope 8.10.0.1 (Veeco)<br>ITC: NanoAnalyzetm software<br>CD: Chirascan 4.5.1848

For manuscripts utilizing custom algorithms or software that are central to the research but not yet described in published literature, software must be made available to editors and reviewers. We strongly encourage code deposition in a community repository (e.g. GitHub). See the Nature Portfolio guidelines for submitting code & software for further information.

Data

Policy information about availability of data

All manuscripts must include a data availability statement. This statement should provide the following information, where applicable:

- Accession codes, unique identifiers, or web links for publicly available datasets
- A description of any restrictions on data availability
- For clinical datasets or third party data, please ensure that the statement adheres to our policy

The data that support the findings of this study are available in this article and it's Supplementary Information. Source data are provided with this paper.

Human research participants

Policy information about studies involving human research participants and Sex and Gender in Research.

Reporting on sex and gender	No human was participated in the research.
Population characteristics	No human was participated in the research.
Recruitment	No human was participated in the research.
Ethics oversight	No human was participated in the research.

Note that full information on the approval of the study protocol must also be provided in the manuscript.

Field-specific reporting

Please select the one below that is the best fit for your research. If you are not sure, read the appropriate sections before making your selection.

[x] Life sciences [ ] Behavioural & social sciences [ ] Ecological, evolutionary & environmental sciences

For a reference copy of the document with all sections, see nature.com/documents/nr-reporting-summary-flat.pdf

Life sciences study design

All studies must disclose on these points even when the disclosure is negative.

Sample size	100 µL of a sample was sufficient for each experimental run. This amount was chosen based on the dimension of the recognition zone of the SERS-CIP.
Data exclusions	No data were excluded from the analyses.
Replication	All of measures were repeated at least three parallel tests to verify the reproducibility of the experimental findings.
Randomization	All of samples were randomly allocated into experimental groups.
Blinding	Characterizations of materials (TEM, SEM, CD, AFM, etc.) and SERS measurements do not routinely use blinded samples since negligible effects from investigators take place.

Reporting for specific materials, systems and methods

We require information from authors about some types of materials, experimental systems and methods used in many studies. Here, indicate whether each material, system or method listed is relevant to your study. If you are not sure if a list item applies to your research, read the appropriate section before selecting a response.

Materials & experimental systems

n/a	Involved in the study
x	Antibodies
x	Eukaryotic cell lines
x	Palaeontology and archaeology
x	Animals and other organisms
x	Clinical data
x	Dual use research of concern

Methods

n/a	Involved in the study
x	ChIP-seq
x	Flow cytometry
x	MRI-based neuroimaging
